# *In silico* screening and molecular analyses identify apigenin from *Scutellaria barbata* as a potent AKT1 inhibitor in breast cancer

**DOI:** 10.1371/journal.pone.0338874

**Published:** 2026-06-25

**Authors:** Md. Maruf Khan, Md. Arju Hossain, Fatematuz Zohora, Md. Sohel, Md. Faisal Amin, Fatematuz Zuhura Evamoni, M. Nazmul Hoque

**Affiliations:** 1 Department of Biochemistry and Molecular Biology, Primeasia University, Dhaka, Bangladesh; 2 Department of Biochemistry and Biotechnology, Khwaja Yunus Ali University, Sirajganj, Bangladesh; 3 Biomedical Engineering, University of Alberta, Edmonton, Alberta, Canada; 4 Department of Biochemistry and Molecular Biology, Mawlana Bhashani Science and Technology University, Tangail, Bangladesh; 5 School of Integrative Biological and Chemical Sciences, The University of Texas Rio Grande Valley, Brownsville, Texas, United States of America; 6 Department of Biotechnology and Genetic Engineering, Noakhali Science and Technology University, Noakhali, Bangladesh; 7 Molecular Biology and Bioinformatics Laboratory, Department of Gynecology, Obstetrics and Reproductive Health, Gazipur Agricultural University, Gazipur, Bangladesh; Hong Kong Baptist University, HONG KONG

## Abstract

Breast cancer (BC) remains a leading cause of cancer-related mortality in women worldwide. *Scutellaria barbata*, a traditional Chinese medicinal herb, possesses recognized anticancer properties, but its mechanistic role in BC is not fully elucidated. This study employed an integrated *in silico* approach to identify the key flavonoids, targets, and pathways through which *S. barbata* exerts its anti-BC effects. A multi-step computational methodology incorporating network pharmacology, molecular docking, and dynamics simulations was utilized to profile bioactive compounds from *S. barbata*. From an initial set of 34 phytocompounds, three flavonoids namely apigenin, 4’-hydroxywogonin, and hispidulin demonstrated favorable drug-likeness, high bioavailability, and low predicted acute oral toxicity (LD₅₀ > 500 mg/kg) profiles. Network analysis identified AKT1, IL6 and TNF as central hub targets, significantly enriched in the PI3K-Akt, MAPK, and TNF signaling pathways. Molecular docking showed strong binding affinities (≤ –7.5 kcal/mol) between these flavonoids and the hub proteins, with hispidulin (–8.1 kcal/mol) and apigenin (–7.7 kcal/mol) exhibiting the highest affinity for AKT1 than other hub proteins. Molecular dynamics simulations over 100 ns further revealed that the apigenin-AKT1 complexes exhibited greater stability with lower RMSD fluctuations, reduced residue flexibility (RMSF), stable radius of gyration, and consistent SASA profiles, compared to other ligands and the control compound. Our computational prediction suggests that apigenin exhibits favorable multi-target interactions with BC-associated proteins. Thus, apigenin may represent a potential candidate for further investigation, particularly with respect to oncogenic signaling pathways involving key hub proteins. However, experimental validation through *in vitro* and *in vivo* studies is required to confirm these observations.

## Introduction

Breast cancer (BC) continues to pose a major global health burden, being the most commonly diagnosed cancer and a leading cause of cancer-related death among women worldwide [1]. Globally, BC ranks second among all cancers, with an estimated 2.3 million cases (11.6%) and 670,000 deaths reported in 2022; notably, 0.5–1% of cases occur in men [2,3]. In the United States, BC incidence rose by 0.5% during 2014–2018 and increased again by 1% between 2012 and 2021, while mortality declined substantially by 44% from 1989 to 2022 [4]. In Europe, approximately 580,000 new breast cancer cases and 160,000 related deaths were reported in 2020 [5]. In contrast, Asia recorded 985,400 new cases and 315,100 deaths in the same year, with projections estimating a rise to 1.4 million cases and 500,000 deaths by 2050 [6]. Molecular heterogeneity underlies BC progression and therapeutic response, with subtypes including HER2-positive, triple-negative, luminal A, and luminal B [7]. HER2-positive BC, characterized by HER2 protein overexpression, accounts for 15–20% of cases [8], while triple-negative BC (TNBC), lacking estrogen, progesterone, and HER2 receptor expression, represents 10–15% of cases [9]. Luminal subtypes are the most prevalent (~70%), defined by hormone receptor positivity and further classified into HER2-negative luminal A and HER2-positive luminal B tumors [10]. Although 5–10% of BC cases are linked to inherited genetic mutations, 20–30% are associated with modifiable lifestyle and environmental risk factors, suggesting that behavioral interventions could substantially reduce disease burden [11].

Conventional BC therapies continue to face major limitations that compromise treatment efficacy and patient well-being. Severe systemic toxicities often damage healthy tissues, resulting in debilitating side effects that reduce quality of life [12]. Moreover, both intrinsic and acquired drug resistance pose persistent challenges, frequently leading to therapeutic failure and disease recurrence [13]. Tumor heterogeneity further complicates treatment, as targeted therapies effective against one molecular pathway may not eliminate other resistant tumor clones, contributing to relapse [14]. Hence, despite advances in targeted and personalized medicine, these limitations underscore the urgent need for safer and more effective therapeutic strategies [12,13]. In this pursuit, natural products have served as an invaluable reservoir of bioactive compounds, with many chemotherapeutic agents being derived from or inspired by plant sources [15,16]. *Scutellaria barbata* D. Don (*S. barbata*), a perennial herb of the *Lamiaceae* family, is widely used in traditional medicine in China and Korea, where it is known as *Ban Zhi Lian* and *Banjiryun*, respectively [17,18]. Historically, it has been applied to treat snake and insect bites, alleviate throat inflammation, and heal traumatic injuries [19]. Recent pharmacological studies have demonstrated its broad therapeutic potential, including anti-cancer, antiviral, anti-inflammatory, antioxidant, antibacterial, immunomodulatory effects, and reversal of epithelial-mesenchymal transition in various BC models [19–21].

The advent of computational biology provides a powerful toolkit to bridge this gap. *In silico* methodologies, including phytochemical mining, network pharmacology, and molecular docking, offer a systematic and efficient strategy to predict drug-target interactions and elucidate complex mechanisms of action from a holistic perspective [16,22–24]. Network pharmacology, in particular, aligns perfectly with the holistic nature of herbal medicine by constructing compound-target-pathway networks to visualize and predict the multi-scale effects of a natural product [25]. This approach enables systematic identification of key molecular targets and signaling pathways, providing mechanistic insights that can guide experimental validation of herbal compounds [26]. In contrast, molecular docking is a computational approach that examines how bioactive compounds interact with target proteins, providing a mechanism for understanding binding orientation, affinity, and the overall stability of the complex. These two methods offer an efficient way to explore potential therapeutic compounds, particularly plant-derived molecules, while reducing reliance on experimental screening and improving early-stage drug discovery success [27].

The selection of *S. barbata* as a source for AKT1-targeting compounds is grounded in its established ethnopharmacological use in traditional Chinese medicine for treating inflammatory diseases and cancers, including breast malignancies. Modern research has corroborated these effects, demonstrating that *S. barbata* extracts exert potent anti-proliferative and pro-apoptotic activities in BC cell lines, including TNBC, through the modulation of key oncogenic pathways such as PI3K/AKT and STAT3 [28,29]. Apigenin, a major bioactive flavone found abundantly in *S. barbata*, has emerged as a promising chemopreventive and therapeutic agent [30,31]. Recent experimental studies have specifically implicated apigenin in the inhibition of the PI3K/AKT/mTOR axis-a critical driver of BC progression and therapy resistance [29,32]. For instance, apigenin was shown to suppress tumor growth and enhance chemosensitivity in HER2-positive and ER-positive BC models by downregulating phospho-AKT levels [33,34]. Furthermore, clinical and epidemiological data suggest an inverse association between dietary apigenin intake and BC risk, highlighting its translational relevance [35]. Therefore, building upon this compelling body of pre-clinical and epidemiological evidence, we hypothesized that apigenin from *S, barbata* could serve as a potent, natural-product-derived AKT1 inhibitor, warranting detailed computational validation and mechanistic exploration in the context of BC. In this study, we employed an integrative *in silico* strategy combining network pharmacology, Gene Ontology (GO) and pathway enrichment analysis, molecular docking, and molecular dynamics simulations to explore the potential molecular mechanisms and target interactions of *S. barbata* against BC. Network pharmacology, a systems-based approach integrating biology, network analysis, and computational modeling, enables the identification of compound-target-pathway relationships and supports mechanism-based drug discovery [25,36]. This study elucidates the molecular mechanisms underlying the anti–BC activity of *S. barbata* and highlights its translational potential in drug discovery. A comprehensive phytochemical library of *S. barbata* was systematically compiled and integrated into a BC-related protein–protein interaction network to identify key dysregulated molecular targets and signaling pathways. Subsequently, a series of *in silico* analyses including pharmacokinetic and toxicity assessment, molecular docking, pharmacophore modeling, molecular dynamics simulations, and quantum mechanical calculations were performed to rigorously evaluate the binding affinity, structural stability, and interaction energetics of the top-ranked flavonoid compounds with BC-associated oncoproteins. Collectively, this integrative and multilayered computational framework bridges traditional ethnopharmacological knowledge with modern computational biology, offering mechanistic insights into the anti-BC potential of *S. barbata*-derived compound apigenin. Furthermore, the findings highlight apigenin as the promising lead compound that warrant subsequent experimental validation and may serve as potential candidate for rational anticancer drug development.

## Materials and methods

### Ethical statement

This research was conducted exclusively through *in silico* methods, including molecular screening and dynamics simulation. As no human subjects, human data, animal experiments, or biological samples were used, ethical approval from an institutional review board was not applicable. The authors affirm that this work adheres to the publication ethics and responsible research practices outlined by PLOS ONE.

### Filtering of the bioactive compounds

To identify potential drug-like molecules from *S. barbata*, its constituent bioactive compounds were sourced from public phytochemical repositories namely Traditional Chinese Medicine Systems Pharmacology Database v3.0 (TCMSP v3.0) [37] and KNApSAcK [38]. Standardized molecular representations such as simplified molecular input line entry system (SMILES), compound identification (CID) number and molecular weight for all compounds were acquired from the PubChem (https://pubchem.ncbi.nlm.nih.gov/) database to ensure computational consistency. A preliminary virtual screening was conducted based on two critical ADME (Absorption, Distribution, Metabolism, and Excretion) parameters: oral bioavailability (OB) and a quantitative estimate of drug-likeness (DL). Compounds with higher OB and DL scores are more likely to be efficiently absorbed into systemic circulation and to exhibit favorable physicochemical properties consistent with established drug-like characteristics [39]. The selection thresholds were set at OB ≥ 30% and DL ≥ 0.18 to focus on compounds with a higher probability of possessing suitable pharmacokinetic properties [40]. Compounds meeting these criteria were subsequently profiled using specialized bioinformatics tools *viz*. the SwissADME platform (http://www.swissadme.ch/) was employed to estimate the bioavailability score while the drug-likeness score was calculated using the Molsoft server (https://www.molsoft.com/mprop/).

### *In silico* pharmacokinetic and toxicity profiling of the screened compounds

A comprehensive *in silico* ADMET (Absorption, Distribution, Metabolism, Excretion and Toxicity) profiling was conducted on the selected compounds. Initially, the SWISSADME web tool (https://www.swissadme.ch/) was utilized to evaluate the physicochemical properties and drug-likeness profiles of the selected compounds. Subsequently, pharmacokinetic parameters (ADME) were predicted using the pkCSM server (https://biosig.lab.uq.edu.au/pkcsm/). Furthermore, the potential toxicological risks of the compounds, such as mutagenicity, carcinogenicity, cytotoxicity, and immunotoxicity, were assessed using the ProTox 3.0 [41] online platform (https://tox.charite.de/protox3/).

### Target prediction of active compounds

Potential protein targets of the bioactive *S. barbata* compounds were predicted using an integrated computational approach. The SwissTargetPrediction platform (http://www.swisstargetprediction.ch/) was employed for its reverse molecular similarity screening. This tool employs a reverse screening approach based on molecular similarity to predict the most likely protein targets of small molecules [42]. Understanding the interactions between proteins and small molecules is crucial for elucidating their molecular and cellular functions. STITCH integrates data from drug–target interactions, binding assays, crystal structures, and metabolic networks, offering a comprehensive view of molecular interactions. These predictions were supplemented and contextualized using the STITCH database (http://stitch.embl.de/), which aggregates drug-target interactions from diverse sources [43], and the Comparative Toxicogenomics Database (CTD), a curated repository of chemical-gene-phenotype-disease relationships [44].

### Identification of BC-associated genes and functional enrichment analysis

BC-related genes were systematically retrieved from publicly available databases, including PubChem, the Comparative Toxicogenomics Database (CTD), Online Mendelian Inheritance in Man (OMIM) [35] and GeneCards [45], using “Breast cancer” as the primary search term. The intersection between these disease-associated genes and targets of active compounds was identified using a Venn diagram tool (https://bioinformatics.psb.ugent.be/webtools/Venn/) to define a core set of genes for subsequent analysis. To elucidate the functional implications of these core genes, enrichment analyses were performed [24,46]. Gene Ontology (GO) annotation was conducted to categorize gene functions across three domains: biological process (BP), cellular component (CC), and molecular function (MF) [47]. Concurrently, Kyoto Encyclopedia of Genes and Genomes (KEGG) pathway analysis was employed to identify significantly enriched signaling and metabolic pathways [23]. Both GO and KEGG analyses were executed using the DAVID bioinformatics resource [48] with *Homo sapiens* as the reference species. Statistically significant terms (p ≤ 0.05) with a high gene count were selected. The results were visualized as enrichment bubble plots using the SRplot web server (http://www.bioinformatics.com.cn/en).

### Construction of protein-protein interaction network and hub protein identification

Protein-protein interaction (PPI) analysis was conducted to elucidate the functional associations among candidate target proteins and to identify key regulatory hubs involved in BC–related pathways. Initially, overlapping target genes shared by apigenin (CID: 5280443), 4′-hydroxywogonin (CID: 5322078), and hispidulin (CID: 5281628) and BC-associated genes were compiled into a unified protein list. These proteins were uploaded to the STRING database (v12.0) [49] with *H. sapiens* selected as the reference organism, and interaction sources were restricted to experimental evidence, curated databases, and predicted interactions, applying a minimum confidence score threshold of >0.4 to ensure reliable associations [24,46]. The generated PPI network was downloaded and imported into Cytoscape (v3.10.3) [50] for visualization and topological analysis, where nodes represent proteins and edges denote physical or functional interactions. Hub proteins were identified using the CytoHubba plugin (https://apps.cytoscape.org/apps/cytohubba) within Cytoscape software [51], employing twelve topological algorithms such as degree (D), maximal clique centrality (MCC), maximum neighborhood component (MNC), betweenness centrality, closeness centrality (CC), edge percolated component (EPC), density of maximum neighborhood component (DMNC), bottleneck, clustering coefficient, eccentricity, radiality, and stress [52,53]. Local-based methods (D, MNC, and MCC) ranked proteins based on direct connectivity, whereas global-based methods (betweenness centrality and CC) ranked proteins according to their overall influence within the network topology [54]. To improve robustness and minimize algorithm-specific bias, the top-ranked genes from all twelve CytoHubba algorithms were integrated, and those appearing in at least nine of the twelve top-ranked lists were designated as hub proteins.

### Construction and visualization of the compound–target–pathway network

To investigate the interconnections among active compounds, their target genes, and the associated biological pathways, a compound–target–pathway (C–T–P) network was constructed using Cytoscape v3.10.4 [51]. Two datasets were imported into Cytoscape: the first included hub proteins and active compounds, represented as target and source nodes, respectively, while the second contained genes and pathways, where genes served as source nodes and pathways as targets. These datasets were integrated using the merge tool of Cytoscape, and the resulting network was visualized by applying various graphical operations such as color coding, layout adjustments, and shape customization to enhance interpretability [24].

### Preparation and energy minimization of ligands and protein structures for molecular docking

The three-dimensional (3D) structures of apigenin (CID: 5280443), 4’-hydroxywogonin (CID: 5322078), hispidulin (CID: 5281628), resveratrol (CID: 445154), tartaric acid (CID: 444305), lenalidomide (CID: 216326), and obatoclax (CID: 45356963) were retrieved from the PubChem database [55]. Among these, apigenin, 4’-hydroxywogonin, and hispidulin were identified as the three most active flavonoids of *S. barbata*, while resveratrol, tartaric acid, lenalidomide, and obatoclax were used as control drugs. The control compounds lenalidomide was used as a reference compound due to its reported *in vivo* antitumor activity mediated by immunomodulatory effects, including suppression of TNF-α [56,57], whereas resveratrol was included as a biologically relevant reference compound based on previously published *in silico* and experimental evidence demonstrating its direct interaction with AKT and its modulatory role via NQO2 [58,59]. In contrast, tartaric acid was selected based on previously published *in silico* studies and its presence in IL-6 crystal structures [60, 61]. Energy minimization of all compounds was performed using the mmff94 force field [62] and the steepest descent optimization algorithm, employing 2000 steps and an energy convergence threshold of <0.01 kcal/mol to stabilize their conformations [62]. Similarly, the 3D crystal structures of AKT1 (PDB ID: 4GV1), IL-6 (PDB ID: 1ALU) and TNF-α (PDB ID: 7JRA) were obtained from the RCSB Protein Data Bank (https://www.rcsb.org/), which offers tools for structural analysis, visualization, and data retrieval [63]. Each PDB file was processed in BIOVIA Discovery Studio [64] to isolate the relevant protein chain and remove water molecules and small ligands, retaining only the co-crystallized drug to preserve the active site architecture. Subsequently, Swiss-PDB Viewer v4.1.0 [65] was used for energy minimization with the GROMOS96 43B1 parameter set. Finally, the co-crystallized ligands were removed through the BIOVIA Discovery Studio to prepare the proteins for subsequent molecular docking analyses.

### Molecular docking analysis

Molecular docking was performed to evaluate the binding affinity and interactions of the lead compounds with key target proteins. Simulations were conducted using AutoDock Vina v1.1.2 integrated into the PyRx v0.8 software platform [65]. The active binding sites for each protein, e.g.*,* AKT1, IL6 and TNF were predicted using the CASTpFold web server [66]. Site-specific rigid docking was carried out by defining grid boxes on the predicted active sites with the following grid box centers AKT1 (X = −20.95Å, Y = 5.23Å, Z = 11.41Å), IL6 (X = 10.17Å, Y = −20.18Å, Z = 16.62Å) and TNF (X = 1.78Å, Y = 3.27Å, Z = −28.25Å) and dimensions AKT1 (X = 29.78Å, Y = 37.38Å, Z = 22.95Å), IL6 (X = 13.31Å, Y = 20.00Å, Z = 19.98Å), and TNF (X = 49.36Å, Y = 26.93Å, Z = 37.93Å). The most favorable docking pose for each ligand-protein complex was selected based on the lowest (most negative) binding energy (ΔG, kcal/mol) and root-mean-square deviation (RMSD) values. An RMSD value ≤ 2.0 Å was considered acceptable, confirming the reliability of the docking protocol [53]. Then, we compared the ligand-protein docking score and non-bonded interactions with the controlled drug. The compounds that had the lowest (most negative) binding energy and good non-bonded interactions compared to the standard drugs were then considered for further analysis. Molecular interactions, including hydrogen bonds and hydrophobic contacts, were visualized and analyzed using UCSF Chimera [67] and BIOVIA Discovery Studio [64].

### Structure based pharmacophore model of ligand

To elucidate the key interaction features of the three bioactive compounds of *S. barbata* namely apigenin (CID: 5280443), 4’-hydroxywogonin (CID: 5322078) and hispidulin (CID: 5281628) with the AKT1 protein (PDB ID: 4GV1), a structure-based pharmacophore analysis was performed using resveratrol (CID: 445154) as the reference ligand. Following molecular docking, protein-ligand complexes were imported into LigandScout 4.4 Advanced (Inte:Ligand GmbH, Austria) [68] to generate pharmacophore models. The software automatically identified key pharmacophoric features including hydrogen bond acceptors (HBA), hydrogen bond donors (HBD), aromatic rings, and hydrophobic regions from the docked conformations. Resulting models were visualized in both 3D and 2D formats to map essential interaction patterns. These pharmacophore profiles were subsequently used to evaluate the spatial and electronic complementarity of each ligand within the AKT1 binding pocket, providing mechanistic insight into their binding modes relative to the reference compound.

### Molecular dynamics simulation of AKT1–ligand complexes

We performed a 100 ns molecular dynamics simulation (MDS) to evaluate the structural stability and dynamic behaviour of the lead compounds such as apigenin, 4’-hydroxywogonin and hispidulin in comparison with the reference molecule resveratrol bound to the AKT1 kinase (PDB ID: 4GV1), a key regulator in BC signalling. Simulations were conducted using Desmond-Maestro v2025.1 Premium (Schrödinger LLC) on a Linux platform, employing the OPLS4 force field for high-fidelity modelling of biomolecular interactions [69]. Protein–ligand complexes were prepared using the Protein Preparation Wizard by assigning pH 7.0, correcting bond orders, adding missing atoms, optimizing hydrogen bonds, and applying restrained minimization to heavy atoms. Systems were solvated in a TIP3P water model within an orthorhombic periodic box with a 10 Å buffer, followed by the addition of 0.15 M Na ⁺ /Cl⁻ ions to ensure physiological ionic strength and charge neutrality. Energy minimization was performed using the steepest descent algorithm for 100 ps. MDS were executed under an NPT ensemble at 310 K using the Nosé–Hoover chain thermostat, with pressure maintained at 1.01325 bar via the Martyna–Tobias–Klein barostat [70]. Trajectory frames were recorded every 100 ps. System stability and binding behaviour were assessed through root mean square deviation (RMSD), root mean square fluctuation (RMSF), solvent-accessible surface area (SASA), radius of gyration (Rg), and hydrogen bond analyses, providing comprehensive insights into ligand stability and conformational flexibility relative to the control molecule resveratrol [23,46,47].

### MM-GBSA binding free energy analysis

We used gmx_MMGBSA tool in combination with GROMACS v23 [71] to examine the binding free energy (ΔG) of the ligand-receptor complex by utilizing the Molecular Mechanics Generalized Born Surface Area (MM-GBSA) techniques. The binding free energy (ΔGbind) was obtained using the following equation:


$$\begin{array}{c} \Delta Gbind\;=\;\Delta EMM\;+\;\Delta Gsolv\;-\;T\Delta S \end{array}$$ (1)

where *ΔE*_MM_ includes van der Waals, Coulombic, covalent, lipophilic, packing, and hydrogen bond interactions. *ΔG*_solv_ comprises polar solvation (GB) energy while entropy *TΔS* was not calculated. The results were averaged across multiple frames to ensure statistical reliability. Negative ΔG values indicate favourable thermodynamically interactions, suggesting strong and stable ligand binding to the target receptor [72].

### Electronic structure and reactivity analysis

All density functional theory (DFT) based computational analyses were conducted using Gaussian v09 [73]. Geometry optimizations and electronic property calculations were conducted by applying the B3LYP functional in combination with the 6-31G(d,p) basis set. GaussView 6.0 [74] was used for input preparation and visualization of molecular structures and electrostatic potential maps [23]. Ligands that showed strong binding affinities in docking and molecular dynamics simulations underwent DFT-based assessment to explore the frontier molecular orbitals (HOMO and LUMO), energy gaps, and molecular electrostatic potential (MEP) for charge distribution [46,47]. The HOMO and LUMO orbital’s energy were then applied to calculate chemical hardness (η) and softness (S) using the following expressions derived from Parr-Pearson theory and Koopmans’ theorem [75].


$$\begin{array}{c} \eta =\frac{\varepsilon_{HOMO-}\varepsilon_{LUMO}\;}{\text{2}} \end{array}$$ (2)

$$\begin{array}{c} s=\frac{\text{1}}{\eta } \end{array}$$ (3)

Greater reactivity is indicated by a lower hardness (η) and higher softness (S) value, while a higher hardness (η) and lower softness (S) values indicate reduced reactivity. Softness (S) measures an atom’s ability to accept electrons and is inversely proportional to hardness (η).

## Results

### Screening and toxicity profiling of *S. barbata* bioactive compounds

A total of 112 potential active compounds from *S. barbata* were initially identified from database mining. For downstream *in silico* analysis, 84 compounds were selected after the exclusion of duplicate entries and compounds with invalid SMILES scores. These 84 compounds were subsequently refined to a final set of 34 prioritized compounds based on favorable bioavailability (score ≥ 0.3) and drug-likeness (score ≥ 0.18) properties. Toxicity profiling of these 34 compounds using the ProTox 3.0 tool revealed that only three compounds namely apigenin, 4’-hydroxywogonin, and hispidulin were classified as non-toxic, whereas the remaining compounds exhibited a range of toxicity endpoints (S1 Table). Immunotoxicity was the most common adverse effect, observed in 15 compounds, including scutebarbatine (A, C, D, E, F, H, I, K, L), 6-O-Acetylscutehenanine A, 5,7,4’-trihydroxy-6-methoxy flavanone, rivularin, scutevulin, salvigenin, and rhamnazin. Five compounds, such as campesterol, beta-sitosterol, stigmasterol, 24-Ethylcholest-4-en-3-one, and sitosterol displayed both neurotoxicity and immunotoxicity. Other significant toxicities identified included carcinogenicity, mutagenicity, and cytotoxicity, with some compounds like ursolic acid and stigmasta-5,22-dien-3-ol-acetate exhibiting multiple toxicity profiles. Moreover, stigmasta-5,22-dien-3-ol-acetate showed neurotoxicity, carcinogenicity, and immunotoxicity in the analysis of toxicity. The ProTox-3 web server predicted acute oral toxicity based on GHS classification criteria. According to the predicted LD₅₀ values, apigenin (2500 mg/kg) was classified as toxicity Class IV (harmful if swallowed; 300 < LD50 ≤ 2000 mg/kg), while 4′-hydroxywogonin (3919 mg/kg) and hispidulin (4000 mg/kg) were classified as Class V (may be harmful if swallowed; 2000 < LD50 ≤ 5000 mg/kg), indicating low acute oral toxicity. All additional data are provided in S1 File.

### Drug-likeness properties of three lead compounds of *S. barbata*

After being confirmed as non-toxic, the three lead compounds *viz*. apigenin, 4’-hydroxywogonin, and hispidulin were further evaluated for their drug-like potential. All three compounds exhibited molecular weights within the acceptable drug-like range (<500 g/mol), with apigenin at 270.24 g/mol, 4’-hydroxywogonin at 300.26 g/mol, and hispidulin at 300.26 g/mol. Their predicted water solubility further indicated that all were highly soluble. Importantly, none of the compounds violated major drug-likeness criteria, including the Lipinski, Veber, Ghose, Muegge, and Egan rules, and each showed a bioavailability score of 0.55. Collectively, these features highlight the strong drug-like potential of the three candidate molecules. Pharmacokinetic profiling further demonstrated that all three compounds possess favorable ADME characteristics. In terms of absorption, apigenin, 4’-hydroxywogonin, and hispidulin showed high predicted intestinal absorption rates of 93.25%, 87.47%, and 84.65%, respectively. Among them, only apigenin displayed high Caco-2 permeability (1.007), while all three exhibited similar skin permeability (logKp = –2.735). Although each compound was identified as a P-glycoprotein substrate, none acted as P-glycoprotein inhibitors. Regarding distribution, apigenin showed comparatively better BBB permeability (–0.734) than 4’-hydroxywogonin (–1.108) and hispidulin (–1.12), yet all three demonstrated CNS penetration potentials within the range of –2.061 to –2.389 (S2 Table). Metabolic predictions indicated that the compounds are primarily processed by cytochrome P450 enzymes, with none serving as CYP2D6 substrates. Apigenin was predicted to be a non-substrate of CYP3A4, in contrast to 4’-hydroxywogonin and hispidulin. All three inhibited CYP1A2 and CYP2C19, while only 4’-hydroxywogonin and hispidulin also inhibited CYP2C9; CYP2D6 and CYP3A4 remained unaffected across all compounds. Finally, the predicted total clearance values (logCLtot) ranged from 0.513 to 0.566 mL/min/kg, and none of the compounds were substrates of renal OCT2, further supporting their favorable pharmacokinetic profiles. All the Physicochemical, drug likeness and pharmacokinetics properties of the three lead compounds of *S. barbata* are shown in S2 Table.

### Identification of genes associated with three lead compounds of *S. barbata* and human BC

To elucidate their potential mechanism of action against BC, the molecular targets of these three compounds were mapped against known BC-associated genes. Initially, a total of 646 human target genes associated with the three selected *S. barbata* compounds were retrieved from SwissTargetPrediction, CTD, and STITCH. After removing duplicates on the basis of gene symbol, 426 unique genes were retained for downstream analyses (S2 File). To identify BC–related genes, multiple databases including GeneCards (15,238), OMIM (300), CTD (122), and PubChem (142) were queried. This search yielded 15,802 genes, which were subsequently screened and duplicate genes were removed based on gene symbol, resulting in 15,291 unique BC–associated genes. The gene sets for the *S. barbata* compounds and BC were then intersected using a Venn diagram, revealing 387 shared genes (S1 Fig and S3 File). These shared targets suggest that *S. barbata* may be associated with breast cancer-related molecular pathways.

### Functional enrichment analysis reveals potential therapeutic effects between *S. barbata* lead compounds and human BC

**Fig 1:**
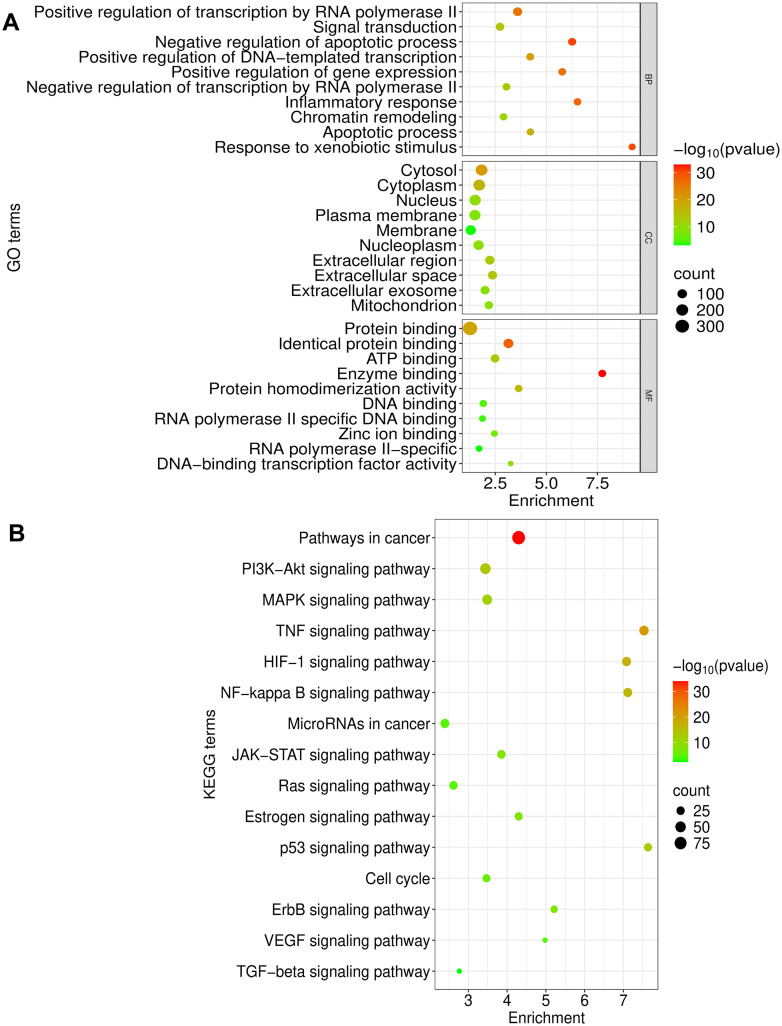
The functional enrichment analysis of shared target genes associated with apigenin, 4’-hydroxywogonin, and hispidulin in breast cancer. **(A)** Gene ontology (GO) enrichment analysis of shared genes categorized into Biological Process (BP), Cellular Component (CC), and Molecular Function (MF). The x-axis displays enrichment scores, while the y-axis lists the corresponding GO terms. Node color represents significance [-log10(p-value)], with red indicating highly significant terms, and node size denotes the number of genes per term. **(B)** Kyoto Encyclopedia of Genes and Genomes (KEGG) pathway enrichment bubble plot illustrating the enriched pathways of shared genes. Enrichment scores are shown on the x-axis, and KEGG pathway names are presented on the y-axis. Node color reflects significance [-log10(p-value)], where red indicates the most significant pathways, and node size corresponds to the number of genes involved in each pathway.

To elucidate the biological effects of the lead compounds (*e.g.,* apigenin, 4’-hydroxywogonin, and hispidulin) in the context of BC, GO and KEGG enrichment analyses were performed. GO terms were categorized into BP, CC, and MF (S4 File). A total of 1,626 GO terms were initially identified, comprising 1,182 BP, 136 CC, and 308 MF terms. After applying significance criteria (p ≤ 0.05) and filtering for higher term counts, 1,314 enriched GO terms were retained, including 950 BP, 113 CC, and 251 MF terms. The top 10 enriched GO terms from each category were visualized in a bubble plot, with fold enrichment displayed along the x-axis and GO terms along the y-axis (Fig 1A). Among BP terms, the most enriched processes included apoptotic process, inflammatory response, negative regulation of apoptosis, response to xenobiotic stimulus, negative regulation of transcription by RNA polymerase II, signal transduction, positive regulation of transcription by RNA polymerase II, chromatin remodeling, positive regulation of DNA-templated transcription, and positive regulation of gene expression. The predominant CC terms were nucleus, membrane, cytosol, plasma membrane, extracellular region, cytoplasm, extracellular exosome, mitochondrion, extracellular space, and nucleoplasm. For MF, the leading terms included protein binding, DNA binding, RNA polymerase II–specific DNA binding, transcription factor activity, enzyme binding, zinc ion binding, ATP binding, RNA polymerase II–specific activity, and protein homodimerization. These enrichment results strongly suggest that the therapeutic effect of apigenin, 4’-hydroxywogonin, and hispidulin against BC is mediated through the modulation of critical biological activities, including programmed cell death, intracellular signaling, and transcriptional regulation.

### Pathway analysis reveals a multi-targeted mechanism of the *S. barbata* lead compounds against human BC

KEGG enrichment analysis was conducted to further elucidate the signaling pathways modulated by apigenin, 4’-hydroxywogonin, and hispidulin in the context of BC. A total of 387 target genes were enriched across 184 KEGG pathways. After applying significance criteria (p ≤ 0.05) and filtering for pathways with higher gene counts, 174 pathways were retained for further interpretation. The top 15 enriched cancer related pathways were visualized using a bubble plot (Fig 1B), where node size indicates the number of associated genes and node color denotes statistical significance from green (lower significance) to red (higher significance). The top 15 KEGG pathways revealed several cancer related pathways associated with the *S. barbata* compounds among these, the three most significantly enriched pathways were PI3K-Akt signaling pathway, MAPK signaling pathway, and TNF signaling pathway. The other oncogenic and cancer-associated pathways were HIF-1, NF-kappa B, JAK-STAT, Ras, Estrogen, p53, ErbB, VEGF and TGF-beta signaling pathway. These enriched pathways highlight the broad regulatory potential of apigenin, 4’-hydroxywogonin, and hispidulin and suggest their involvement in multiple cancer-associated biological processes relevant to BC therapy. All the additional data related to KEGG pathways are provided in S5 File.

### Building protein-protein interaction network and identification of hub proteins

**Fig 2:**
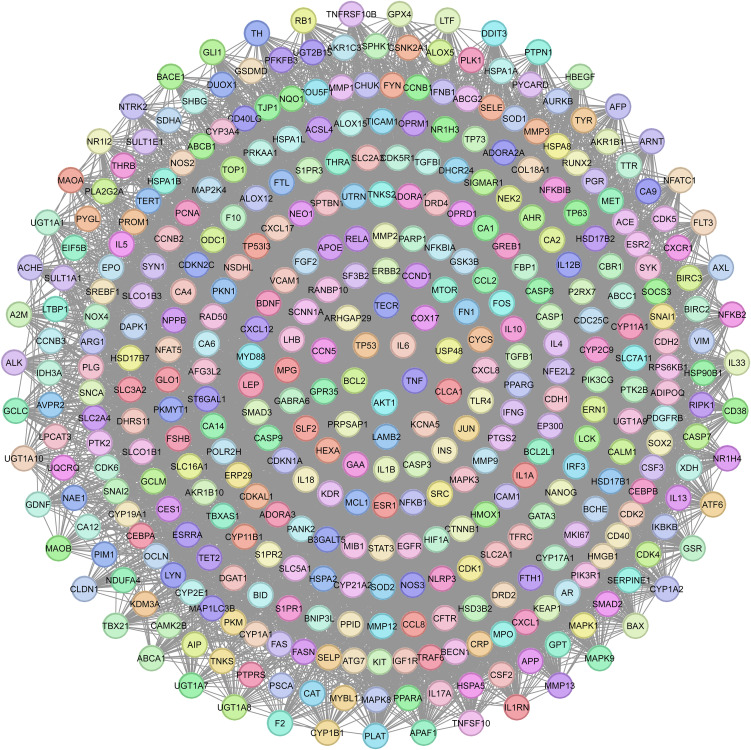
Protein–protein interaction (PPI) network illustrating the potential therapeutic targets of apigenin, 4’-hydroxywogonin, and hispidulin in breast cancer. In this network, proteins are represented as nodes and their interactions as edges. Each protein is shown as an elliptical node, providing a clearer and more intuitive visualization of the interaction patterns.

**Fig 3:**
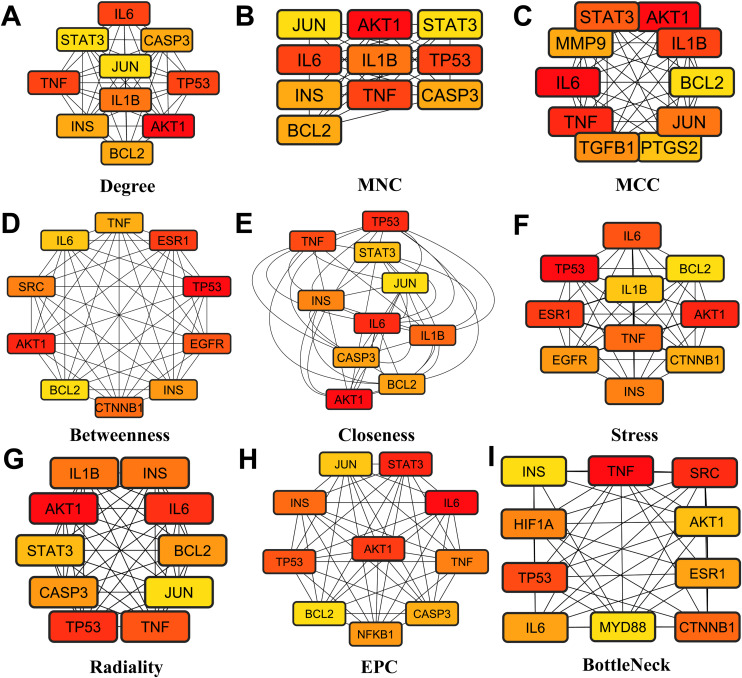
Visualization of the principal hub protein targets associated with apigenin, 4’-hydroxywogonin, and hispidulin in breast cancer, as identified using nine CytoHubba ranking algorithms: **(A)** Degree, **(B)** maximum neighborhood component (MNC), **(C)** maximal clique centrality (MCC), **(D)** Betweenness, **(E)** Closeness, **(F)** Stress, **(G)** Radiality, **(H)** edge percolated component (EPC), and **(I)** BottleNeck.

A PPI network was constructed to elucidate the functional relationships among the target proteins. The PPI network of 387 shared and enriched genes comprised 382 nodes and 11,005 edges, with a network density of 0.076 and a clustering coefficient of 0.290 (Fig 2). In the PPI network, each protein is depicted as an elliptical node, representing an individual protein and enabling a more intuitive interpretation of the interaction patterns. To identify key regulatory proteins, 12 cytoHubba algorithms such as MNC, Betweenness, MCC, Degree, Closeness, Stress, Radiality, EPC, BottleNeck, DMNC, EcCentricity and Clustering Coefficient method were applied (Fig 3 and S6 File). Here, protein appearing in ≥9 among top 12 lists were considered as hubs protein. Based on this consensus criterion, AKT1, IL6, and TNF consistently emerged as hubs (Fig 3 and S2 Fig). Their detailed topological metrics are provided in S3 Table.

### Integrative network analysis reveals core signaling pathways

**Fig 4:**
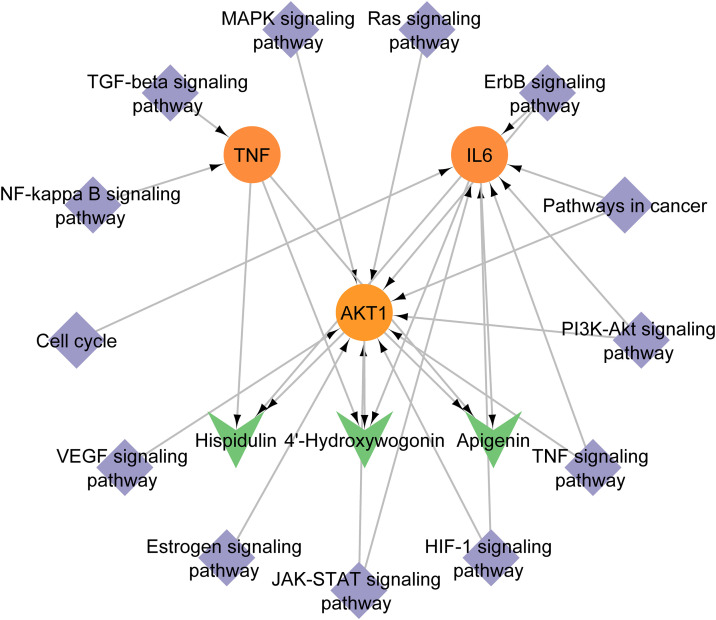
Compound–target–pathway interaction network of the bioactive compounds of *Scutellaria barbata* and hub proteins. Hub proteins (IL6, TNF, and AKT1) are represented as orange circles, the active compounds apigenin, 4’-hydroxywogonin, and hispidulin are shown as green V-shaped nodes, and the associated pathways connected to these targets are depicted as purple diamond-shaped nodes.

To synthesize the findings into a cohesive mechanism, an integrative compound-target-pathway (C-T-P) network was constructed. This network interconnected the three lead compounds, the three core hub proteins (AKT1, IL6 and TNF), and the 13 most significant KEGG pathways associated with these hubs. The resulting network (Fig 4) comprised 22 nodes and 40 edges, graphically depicting the multi-scale therapeutic strategy. The network revealed that the PI3K–AKT signaling pathway, MAPK signaling pathway, HIF-1 signaling pathway and TNF signaling pathway were the most prominent pathways linking the compounds to their gene targets, highlighting their potential importance in mediating the therapeutic effects of the *S. barbata* constituents against BC through their interaction with the core hub proteins.

### Molecular docking reveals strong binding of flavonoids to key oncoproteins

**Fig 5:**
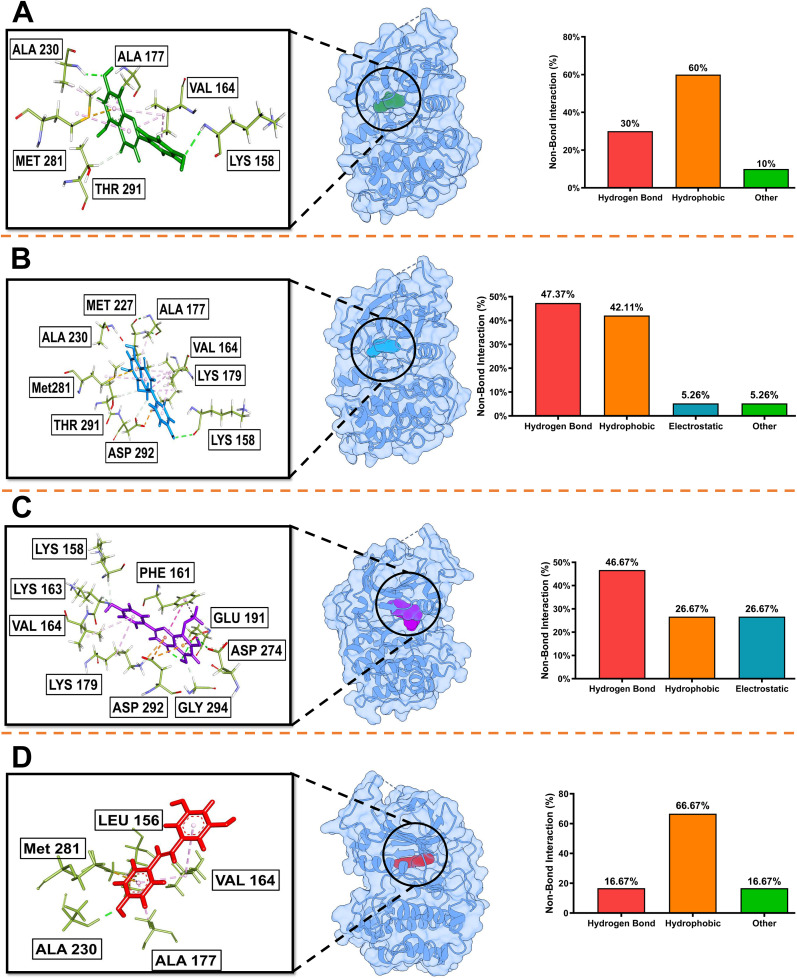
Molecular docking results showing the non-bonding interactions between AKT1 and the studied compounds. The two-dimensional (2D) interaction profiles for **(A)** apigenin, **(B)** 4’-hydroxywogonin, **(C)** hispidulin, and **(D)** resveratrol (control) detail the participating amino acids and the percentage contribution of hydrogen bonds, hydrophobic, electrostatic, and other interactions. The comparative analysis identifies conserved residues critical for binding and unique interactions that may explain differential efficacies among the ligands.

We performed molecular docking analyses to evaluate the binding potential of apigenin, 4′-hydroxywogonin, and hispidulin with key BC-associated target proteins, including AKT1, IL6 and TNF. Docking scores were used to assess ligand–protein binding affinity, where lower binding energies indicate stronger and more stable molecular interactions. Among the screened phytocompounds, hispidulin exhibited the most favorable binding interaction with AKT1, as evidenced by the lowest docking energy (–8.1 kcal/mol). This binding affinity exceeded that of the reference ligand resveratrol (–7.2 kcal/mol) and other candidate compounds, including apigenin (–7.7 kcal/mol) and 4′-hydroxywogonin (–7.6 kcal/mol). For IL6, apigenin showed the highest affinity (–5.9 kcal/mol), followed by 4′-hydroxywogonin (–5.8 kcal/mol) and hispidulin (–5.8 kcal/mol), whereas the control tartaric acid demonstrated weaker binding (–3.9 kcal/mol) (Table 1). Against TNF, apigenin again performed best (–6.5 kcal/mol), exceeding the affinities of 4′-hydroxywogonin (–6.2 kcal/mol), hispidulin (–6.4 kcal/mol), and the control lenalidomide (–6.0 kcal/mol). All three phytocompounds docked within the primary active pocket of each target protein. Notably, all ligands exhibited strong binding to AKT1, with consistently lower binding energies than those observed for the other protein complexes. Apigenin formed hydrogen bonds with LYS158, ALA230, and THR291, along with hydrophobic interactions involving VAL164, MET281, ALA177, and ALA230 (Fig 5A and Table 1). In contrast, 4′-hydroxywogonin established six hydrogen bonds (LYS158, THR291, ASP292, LYS179, ALA177, MET227) and five hydrophobic contacts (VAL164, LYS179, MET227, MET281, ALA177) (Fig 5B and Table 1). Hispidulin formed six hydrogen bonds (GLU191, ASP274, LYS158, LYS163, GLY294, LYS179), three hydrophobic interactions (PHE161, VAL164, LYS179), and three electrostatic contacts (GLU191, ASP292, LYS179) (Fig 5C and Table 1). The control ligand resveratrol engaged AKT1 primarily through a hydrogen bond with ALA230 and hydrophobic interactions with VAL164, LEU156, and ALA177 (Fig 5D). Binding scores and non-bonded interactions of AKT1, IL-6 and TNF are presented in S4 Table. Collectively, these findings indicate that apigenin, 4′-hydroxywogonin, and hispidulin demonstrate promising binding interactions with BC-related targets and may serve as potential therapeutic candidates.

**Table 1:** Docking scores and non-bond interactions of the phytocompounds (e.g., apigenin, 4’-hydroxywogonin, and hispidulin) of *Scutellaria barbata* against AKT1 hub protein in human breast cancer.

Proteins (PDB ID)	Phytocompound	PubChem CID	Docking Score (kcal/mol)	Hydrogen bond Interaction	Hydrophobic interaction
				Residues involved	Residues involved
AKT1 (4GV1)	Apigenin	5280443	−7.7	LYS158, ALA230, THR291	MET281, VAL164, ALA177, and ALA230
	4’-Hydroxywogonin	5322078	−7.6	LYS158, THR291, ASP292, LYS179, ALA177, and MET227	VAL164, LYS179, MET227, MET281, and ALA177
	Hispidulin	5281628	−8.1	GLU191, ASP274, LYS158, LYS163, GLY294, and LYS179	PHE161, VAL164, and LYS179
	Resveratrol (control)	445154	−7.2	ALA230	VAL164, LEU156, and ALA177

### Structure-based pharmacophore mapping and comparative interaction profiling of candidate ligands

Structure-based pharmacophore analysis revealed distinct interaction profiles for the candidate ligands relative to the control compound. Apigenin (S3A Fig) exhibited multiple hydrogen bond donors and acceptors targeting Ala230A, Asp292A, Thr291A, and Met227A, complemented by π–π stacking with Val164A, indicating optimal binding orientation. Hispidulin (S3B Fig) formed fewer hydrogen bonds but engaged key hydrophobic residues (Val164A, Leu181A) and formed electrostatic interactions with Glu191A, suggesting an alternative stabilization mechanism. 4’-hydroxywogonin (S3C Fig) displayed a dense hydrogen-bonding network with Met227A, Lys158A, and Ala230A, alongside π-interactions, yielding a pharmacophore model highly congruent with that of the control drug. In contrast, the control drug resveratrol (S3D Fig) maintained canonical interactions with residues Glu228A, Thr291A, and Lys158A, supported by a hydrophobic ring stacking with Met281A and Leu156A consistent with its established mechanism of inhibition.

### Molecular dynamics simulations reveal structural stability of protein-ligand complexes

**Fig 6:**
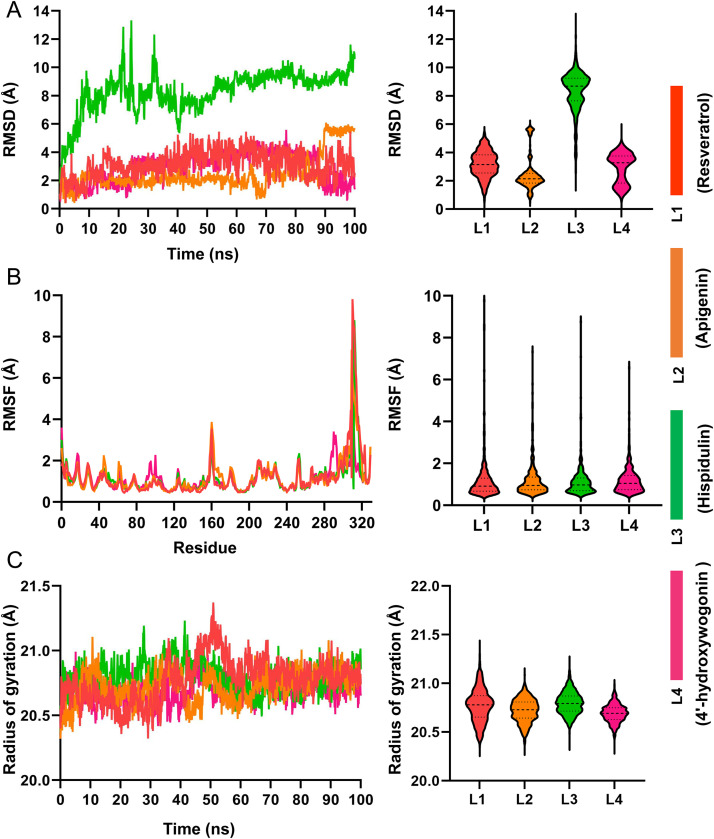
Molecular dynamics stability analysis of AKT1-ligand complexes over 100 ns. **(A)** Ligand- root mean square deviation (RMSD), **(B)** residue-wise root mean square fluctuation (RMSF), and **(C)** radius of gyration (Rg) shown as time-dependent plots (left) and corresponding violin plot distributions (right). L1-L4 represent AKT1 in complex with resveratrol (L1), apigenin (L2), hispidulin (L3), and 4′-hydroxywogonin (L4). Violin plots represent the probability density distribution of each parameter over the simulation, highlighting median values and variability. RMSD indicates overall stability, RMSF reflects residue flexibility, and Rg describes protein compactness.

To assess the dynamic stability and conformational behavior of the protein–ligand interactions, 100 ns MDSs were performed for the AKT1 protein complexed with the three test ligands apigenin, 4’-hydroxywogonin, and hispidulin as well as the control molecule, resveratrol. We selected only AKT1 protein for MD simulation among other hub proteins (IL6 and TNF) due to its highest binding affinity with the ligands. The structural and stability parameters obtained from these simulations were assessed by using RMSD, Rg, RMSF, and SASA. RMSD analysis revealed that the protein–ligand complexes maintained varying levels of structural stability over the 100 ns simulation (Fig 6A). The mean ligand-RMSD (mean ± SD) values recorded were 2.467 ± 1.202 Å for apigenin, 8.318 ± 1.464 Å for hispidulin, 2.912 ± 1.050 Å for 4’-hydroxywogonin, and 3.172 ± 0.9063 Å for the control drug, resveratrol. Peak ligand-RMSD values were observed at 90.3 ns (6.024 Å), 24.3 ns (13.26Å), 76.8 ns (5.513 Å), and 54 ns (5.384Å), while the minimum ligand-RMSD values ranged between 0.455 and 1.858 Å for all test ligands and at 0.484 Å for the control. Hispidulin and the control compound exhibited the greatest structural fluctuations, whereas apigenin and 4’-hydroxywogonin consistently remained below 3.0 Å, indicating comparatively more stable complex formation (Fig 6A). RMSF analysis revealed comparable fluctuation profiles across most residues for all four ligand-bound complexes (Fig 6B). The mean RMSF values were 1.193 ± 0.8214 Å for apigenin-AKT1, 1.135 ± 0.7890 Å for hispidulin-AKT1, 1.184 ± 0.6500 Å for 4’-hydroxywogonin-AKT1, and 1.190 ± 1.047Å for the control-AKT1 complex. Notable peak fluctuations occurred at THR160, ALA171, ALA188, ASN204, GLU267, GLY303, ASP323, GLU359, SER396, GLY410, and SER457. The highest RMSF values were recorded at THR450 for apigenin (7.326 Å), CYS460 for hispidulin (8.758 Å), SER457 for 4’-hydroxywogonin (6.562 Å), and MET458 for the control (9.773 Å). The lowest fluctuations ranged between 0.462–0.466 Å at VAL258 for the test ligands, and 0.453 Å at LEU210 for the control (Fig 6B).

The Rg value for all complexes remained within 20.0-21.5 Å (Fig 6C). The control compound exhibited noticeable fluctuations between 40–60 ns, reaching up to 21.5 Å. Apigenin and hispidulin maintained relatively stable values around 20.5 Å, whereas 4’-hydroxywogonin displayed the lowest and most stable Rg profile, particularly after 50 ns (Fig 6C).

**Fig 7:**
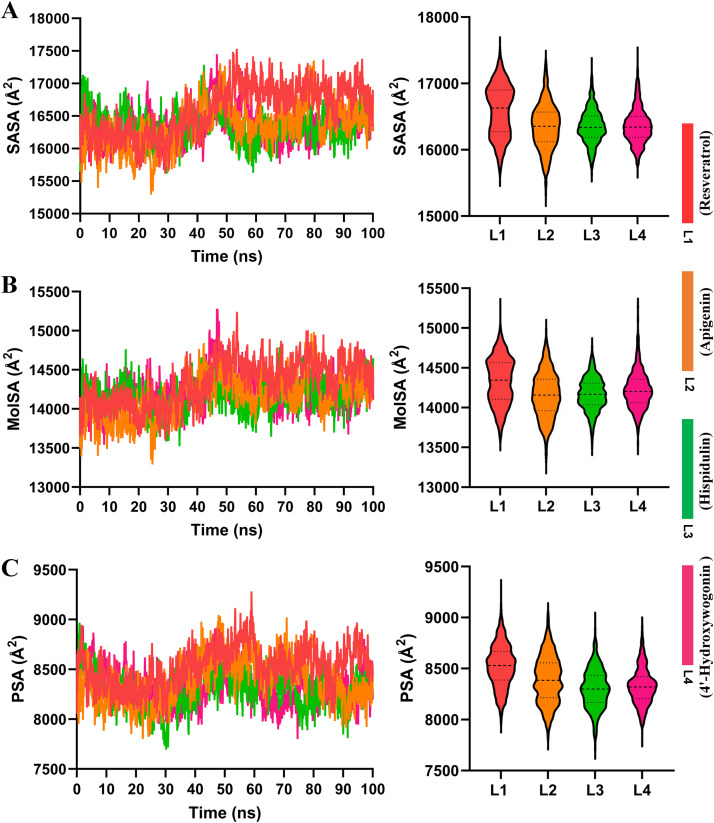
Surface property analysis of AKT1-ligand complexes during a 100 ns molecular dynamics simulation. **(A)** Solvent-accessible surface area (SASA), **(B)** molecular surface area (MolSA), and **(C)** polar surface area (PSA) shown as time-dependent plots (left) and corresponding violin plot distributions (right). L1-L4 represent AKT1 in complex with resveratrol (L1), apigenin (L2), hispidulin (L3), and 4′-hydroxywogonin (L4). Violin plots depict the probability density and variability of each surface parameter over the simulation, emphasizing median behavior. SASA reflects protein surface exposure, MolSA indicates overall surface compactness, and PSA represents the contribution of polar regions during ligand binding.

The SASA profile of all complexes ranged between 15,500 and 17,500Å² for all complexes (Fig 7A). Hispidulin exhibited high fluctuation during 0–30 ns, followed by stabilization. The control showed moderate but consistent variation. Apigenin demonstrated lower solvent exposure than the control, and 4’-hydroxywogonin remained within a similar SASA range as apigenin and hispidulin (Fig 7A). MolSA values indicated varying ligand–protein compactness (Fig 7B). The control showed the highest fluctuation (13,800−15,200Å²). Apigenin stabilized after 40 ns, whereas hispidulin showed the lowest and most consistent values. 4’-hydroxywogonin fluctuated early but stabilized after 50 ns around 14,400Å² (Fig 7B). PSA values for all ligands and the control remained stable between 8000 and 9000Å throughout the simulation (Fig 7C), suggesting a consistent polar surface exposure.

### Dynamic interaction profiles and binding stability of ligand-AKT1 complexes

To evaluate the stability and binding behavior of the selected phytocompounds with AKT1, protein–ligand interactions were monitored over the 100 ns MDS using hydrogen bonds, ionic interactions, hydrophobic contacts, and water-mediated bridges (S4 Fig). Apigenin (S4A Fig) maintained stable interactions primarily through hydrogen bonds with GLU228 and ALA230, while 4’-hydroxywogonin (S4B Fig) formed a more diverse interaction network involving GLU228, ASP292, and ALA230, supplemented by water-bridged hydrogen bonds. In contrast, hispidulin showed a simpler binding profile, predominantly engaging ALA230 (S4C Fig). The control compound resveratrol (S4D Fig) demonstrated interactions with GLU228, VAL164, THR211, and ALA230, stabilized by two structural water molecules. All ligands maintained consistent interaction patterns throughout the simulation, with hydrogen bonds serving as the primary stabilizing force. Apigenin and hispidulin exhibited the most complex interaction profiles, featuring multiple simultaneous hydrogen bonds complemented by hydrophobic and ionic contacts. These detailed interaction maps, visualized through simulation interaction diagrams in S5 Fig, confirm that the candidate ligands form stable, specific complexes with AKT1, with binding modes comparable to or exceeding the stability of the control compound.

### MM-GBSA binding energy decomposition reveals key interactions in AKT1-ligand complexes

**Fig 8:**
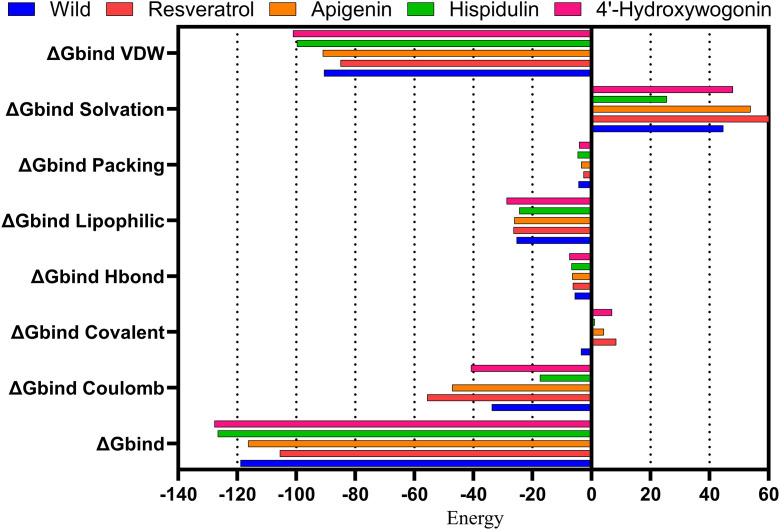
Post simulation thermal molecular mechanics generalized born surface area (MM-GBSA) binding energy decomposition profiles for AKT1 in complex with apigenin, 4’-hydroxywogonin, hispidulin, and the control compound resveratrol. Negative energy contributions indicate favorable (stabilizing) interactions that enhance binding affinity, whereas positive values correspond to unfavorable (destabilizing) interactions within the protein-ligand complexes.

MM-GBSA binding free energy decomposition elucidated the energetic determinants of ligand binding to AKT1 (Fig 8). Hispidulin demonstrated the strongest binding affinity (–127.55 kcal/mol), closely followed by 4’-hydroxywogonin (–125.22 kcal/mol), with both outperforming the control system (–118.91 kcal/mol). Van der Waals interactions constituted the primary stabilizing component across all complexes (–85.05 to –102.01 kcal/mol), with 4’-hydroxywogonin exhibiting the most favorable contribution. Lipophilic interactions provided substantial stabilization (–25 to –28 kcal/mol), while hydrogen bonding ranged from –5.72 to –7.84 kcal/mol, with 4’-hydroxywogonin forming the most stable network. Notably, all ligands incurred significant desolvation penalties, particularly the control compound (+62.25 kcal/mol) (Fig 8). The robust van der Waals and lipophilic contributions effectively compensated for unfavorable solvation energies, confirming the strong binding potential of hispidulin and 4’-hydroxywogonin.

### Density functional theory (DFT) analysis of electronic properties and molecular reactivity

**Fig 9:**
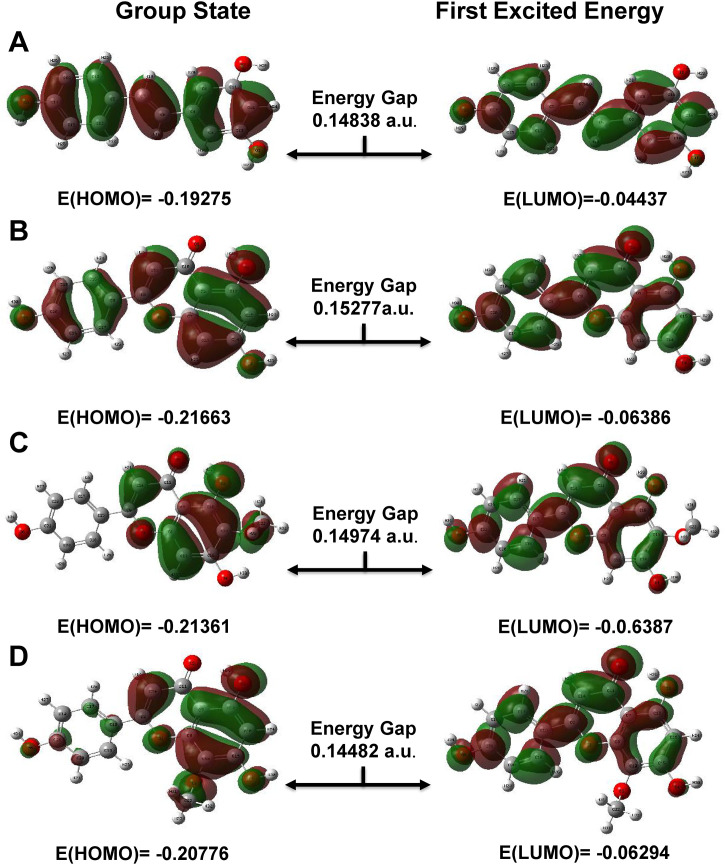
Depiction of the highest occupied molecular orbital (HOMO) and lowest unoccupied molecular orbital (LUMO) energies, along with the structural representations of (A) apigenin, (B) 4’-hydroxywogonin, (C) hispidulin, and (D) the control compound resveratrol, illustrating the electronic distribution and potential reactivity of each ligand.

**Fig 10:**
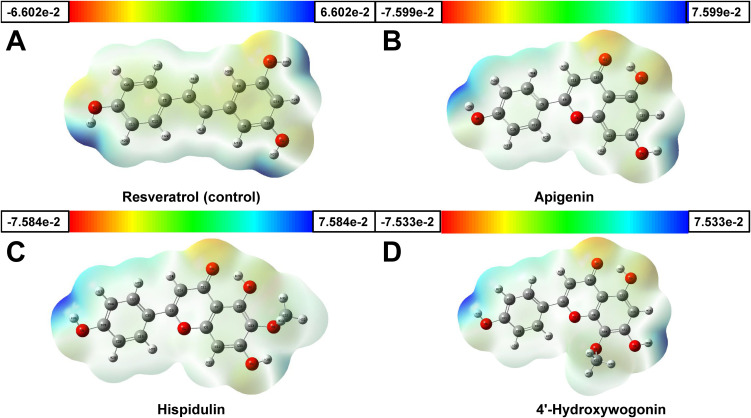
Molecular electrostatic potential (MEP) surfaces of (A) resveratrol, (B) apigenin, (C) hispidulin, and (D) 4′-hydroxywogonin, visualized at the 0.001 e bohr ⁻ ³ isosurface. Regions colored red indicate electron-rich areas, typically acting as hydrogen bond acceptors, while blue regions indicate electron-deficient areas, representing potential hydrogen bond donors, providing insight into the reactive sites and intermolecular interaction potential of each compound.

DFT calculations were conducted to evaluate the electronic properties and chemical reactivity of the flavonoid compounds relative to the control. Key descriptors, including the HOMO–LUMO energy gap, hardness, and softness, are summarized in S5 Table and shown in Fig 9A–9D. The control compound displayed a HOMO–LUMO gap of 0.14838 a.u., along with hardness (*η*) of 0.07419 a.u. and softness (*S*) of 13.47 a.u., serving as the reference benchmark. Among the test ligands, apigenin exhibited the highest HOMO–LUMO gap (0.15277 a.u.) and hardness (0.07638 a.u.), with a slightly reduced softness (13.09 a.u.), indicating marginally lower reactivity than the control. Hispidulin showed a similar electronic profile, with a gap of 0.14974 a.u., hardness of 0.07487 a.u., and softness of 13.35 a.u., suggesting comparable reactivity. In contrast, 4′-hydroxywogonin showed the lowest energy gap (0.14482 a.u.) and hardness (0.07241 a.u.), resulting in the highest softness value (13.81 a.u.), surpassing even the control and indicating enhanced chemical reactivity. These observations align with DFT principles, where reduced hardness and increased softness correspond to greater molecular reactivity. MEP surface analysis further revealed distinct charge distribution across the ligands (Fig 10A–10D). Control compound resveratrol displayed symmetric electron-rich regions on both phenyl rings. Apigenin showed a continuous electronegative surface across the 5-, 7-, and 4′-hydroxyl groups and the carbonyl oxygen. Hispidulin exhibited intensified negative potential near the carbonyl group, attributed to the electron-donating 6-methoxy group. The most pronounced charge separation was observed in 4′-hydroxywogonin, with distinct electron-rich (red) and electron-deficient (blue) regions, reflecting a strong dipole moment. Collectively, the MEP patterns suggest that these functional groups are favorably positioned to interact with AKT1 through hydrogen bonding and electrostatic interactions, supporting their potential role in stabilizing ligand–protein binding.

## Discussion

BC continues to present substantial therapeutic challenges, largely due to its biological heterogeneity and the role of BC stem cells (BCSCs) to tumor progression, metastasis, and disease recurrence [76]. Although standard treatment modalities such as surgery, radiotherapy, and chemotherapy have led to improvements in clinical outcomes, issues related to therapeutic resistance and distant metastasis remain unresolved [1,77]. In recent years, natural products have gained attention as valuable sources of anticancer agents, with nearly 60% of currently used anticancer drugs derived from natural compounds or their analogues [78]. Notably, *S. barbata*, a traditional Chinese medicinal herb, has shown anticancer activity across multiple cancer types, including breast, lung, and gastrointestinal malignancies [17,79].

This integrated computational approach identified three key flavonoids from *S. barbata* including apigenin, 4’-hydroxywogonin, and hispidulin as multi-target agents against BC through comprehensive analysis of their drug-likeness, network pharmacology, molecular interactions and stability analysis. These compounds exhibited excellent *in silico* pharmacokinetic properties, complying with Lipinski’s rule of five and demonstrating favorable ADME characteristics. Their molecular weights below 500 g/mol and optimal water solubility suggest potential for oral bioavailability, a crucial consideration for clinical translation [66]. Computational toxicity analysis placed all three compounds in toxicity classes IV and V, with predicted LD₅₀ values above 2500 mg/kg, implying a relatively low toxicity potential based on *in silico* evaluation. Apigenin has shown low acute toxicity in OECD-425 rat studies with LD₅₀ > 2000 mg/kg [80], while 4’-hydroxywogonin demonstrates low cytotoxicity *in vitro* [81], and hispidulin exhibits reno-protective effects in murine models [82]. Furthermore, *in silico* toxicity assessment predicted no neurotoxic, immunotoxic, hepatotoxic, mutagenic, or carcinogenic liabilities for any of the compounds, highlighting their computationally inferred safety [83].

Network pharmacology analysis revealed the multi-target nature of these flavonoids, with significant enrichment in critical cancer-related pathways. Gene Ontology (GO) analysis identified key biological processes including signal transduction and positive regulation of transcription by RNA polymerase II, while KEGG pathway analysis demonstrated significant enrichment in pathways in cancer, MAPK signaling, TNF signaling, and PI3K-Akt signaling pathways. The PI3K-Akt pathway, frequently hyperactivated in BC, plays crucial roles in cell survival, proliferation, and metabolism [84]. Similarly, MAPK signaling regulates apoptosis, differentiation, and proliferation through various kinase cascades, with dysregulation contributing to carcinogenesis [85]. The TNF signaling pathway, mediated by TNF-α, modulates inflammation and tumor progression, with overexpression correlating with aggressive BC phenotypes and poor prognosis [86]. PPI network and hub gene analyses identified AKT1, IL-6 and TNF as key targets for these flavonoids. AKT1, a central mediator of the PI3K/Akt pathway, is frequently hyperactivated in BC, contributing to tumor development and drug resistance [87]. IL-6, involved in angiogenesis and metastasis, shows elevated expression in breast tumors [88], while TNF-α enhances cancer cell survival and invasion [89].

Molecular docking studies demonstrated strong binding affinities of all three flavonoids to these target proteins. Hispidulin and apigenin showed superior binding affinity to AKT1 (−8.1 kcal/mol and −7.7 kcal/mol) compared to the reference compound resveratrol (−7.2 kcal/mol), while apigenin exhibited the strongest binding to TNF (−6.5 kcal/mol) and IL-6 (−5.9 kcal/mol). Previous *in silico* study reported that AKT inhibitors like capivasertib showed good binding affinity towards AKT1 (−9.5 kcal/mol) and AKT2 (−8.4) [90], while ipatasertib a well-known AKT inhibitor significantly bind with AKT isoforms, particularly AKT2 protein with a docking score of −7.2 kcal/mol [91], suggesting our finding ligands fall within comparable range, supporting their potential AKT-modulatory activity. Detailed interaction analysis revealed that these compounds form multiple hydrogen bonds and hydrophobic interactions with key residues, including GLU228, ALA230, and LYS158 in AKT1, which are critical for kinase inhibition [15,92]. Research demonstrated that hydrogen bonds provide directional and specific contacts that facilitate accurate ligand positioning within the active site, while hydrophobic interactions enhance complex stability by promoting burial of nonpolar surfaces and favorable entropy changes. An optimal balance between these interactions is critical for achieving strong binding, selectivity, and favorable drug-like properties during rational drug design [93]. Pharmacophore modeling provided further insights into the binding characteristics, with 4’-hydroxywogonin showing the most favorable interaction pattern, forming stable hydrogen bonds and aromatic stacking similar to known AKT1 inhibitors. Apigenin displayed moderate polar contacts, while hispidulin relied mainly on hydrophobic interactions, suggesting different binding mechanisms that could be exploited for structure-based drug design [16,94].

MDSs simulation over 100 ns provided a further dynamic assessment of binding stability. Research demonstrated that lower RMSD values indicate that the protein-ligand complexes remained structurally stable throughout the simulation while reduced RMSF values further suggest that ligand binding limited residue-level flexibility, reflecting enhanced local stability of the protein structure. In our study, the apigenin RMSD remained low at 2.467 ± 1.202 Å, indicating minimal structural deviation, while the mean RMSF (1.193 ± 0.821 Å) reflected limited residue-level fluctuations and enhanced local stability. In addition, lower Rg and SASA values indicated that the protein maintained a compact conformation, with tighter packing and stable folding. Our analysis showed that Rg remained consistently around 20.5 Å, suggesting sustained protein compactness, and SASA values were comparatively lower, ranging within 15,500–16,000 Å², indicating reduced solvent exposure and tighter folding. Together, apigenin-AKT1 complex showed a stable Rg and SASA value, suggesting its structural compactness and minimal unfolding nature. Protein-ligand interaction analysis demonstrated stable hydrogen bonds and hydrophobic contacts throughout the simulations, particularly involving key residues GLU228 and ALA230 in AKT1 [94,95]. Based on its stable binding and favorable dynamic interactions with AKT1, apigenin could be considered a promising therapeutic candidate for targeting AKT1-mediated signaling pathways.

MM/GBSA binding free energy calculations provided quantitative assessment of binding affinities, with hispidulin (−127.55 kcal/mol) and 4’-hydroxywogonin (−125.22 kcal/mol) demonstrating superior binding compared to the control system (−118.91 kcal/mol). Van der Waals interactions constituted the primary stabilizing component, while lipophilic interactions provided substantial stabilization. Although all ligands incurred desolvation penalties, the robust van der Waals and lipophilic contributions effectively compensated for these unfavorable energies, confirming strong binding potential [72]. DFT analysis revealed favorable electronic properties for all compounds. The HOMO-LUMO energy gaps, hardness (η), and softness (S) values indicated good chemical reactivity, with 4’-hydroxywogonin showing the smallest gap (0.14482 a.u.), lowest hardness (0.07241 a.u.), and highest softness (13.81 a.u.), suggesting enhanced reactivity. Moreover, the MEP surfaces revealed distinct charge distribution patterns across the compounds. Apigenin exhibited extended electronegative regions around its hydroxyl and carbonyl groups, whereas hispidulin exhibited a more concentrated negative potential near the carbonyl moiety. In contrast, 4′-hydroxywogonin showed the greatest charge separation, indicative of strong dipole moments that could enhance hydrogen bonding and electrostatic interactions [96].

While these computational findings are highly promising, several limitations must be acknowledged. MDSs were performed only for AKT1, which was prioritized due to its favorable docking interactions with the selected ligands. Future studies should extend MD simulations to other hub proteins, such as IL6 and TNF, to provide a more comprehensive understanding of the dynamic behavior of all key targets.

While this study provides a robust computational foundation, its findings remain preliminary as they are derived exclusively from *in silico* methods without experimental validation. The MDS were performed only for AKT1, leaving interactions with other key hub proteins (IL6 and TNF) unexplored. Additionally, network pharmacology relies on static databases that cannot fully capture the dynamic tumor microenvironment. Given the heterogeneity of BC subtypes, the possibility of unanticipated off-target effects cannot be excluded. Consequently, experimental confirmation is required, including *in vitro* assays to evaluate cytotoxicity, apoptosis induction, and modulation of the PI3K/AKT signaling pathway, followed by *in vivo* studies to assess therapeutic efficacy, toxicity, and pharmacokinetic profiles. Further investigations employing patient-derived organoids, appropriate animal models, and combination strategies with established drugs will be necessary to confirm therapeutic potential and to support optimization and translational development.

## Conclusion

This study employed an integrated *in silico* approach to evaluate the anticancer potential of major *S. barbata* phytochemicals, including apigenin, 4′-hydroxywogonin, and hispidulin, against BC. Network pharmacology identified AKT1, IL6, and TNF as key targets, with PI3K-AKT, MAPK, and TNF signaling pathways implicated in their potential mechanisms of action. Molecular docking and 100 ns dynamics simulations suggested stable and favorable binding interactions, particularly for apigenin, which showed the lowest RMSD, reduced RMSF, compact SASA, and favorable Rg values with AKT1. *In silico* ADMET and toxicity predictions further indicated acceptable drug-likeness and safety profiles. Taken together, our *in silico* findings propose apigenin as a promising AKT1-targeting inhibitor for BC, emphasizing the need for further *in vitro* and *in vivo* studies to unveil its full spectrum of biological activity.

## Supporting information

S1 Table Toxicological properties of bioactive compounds (e.g., apigenin, 4’-hydroxywogonin, and hispidulin) of *Scutellaria barbata.* (DOCX)

S2 Table Physicochemical, drug likeness and pharmacokinetics properties of bioactive compounds (e.g., apigenin, 4’-hydroxywogonin, and hispidulin) of *Scutellaria barbata.* (DOCX)

S3 Table Topological characteristics of identified three hub proteins. (DOCX)

S4 Table Docking score and non-bond interactions of apigenin, 4’-hydroxywogonin, and hispidulin against selected IL6 and TNF hub proteins of breast cancer. (DOCX)

S5 Table Energy of highest occupied molecular orbitals (HOMOs), lowest unoccupied molecular orbitals (LUMOs), gaps, hardness, and softness of phytocompounds (e.g., apigenin, 4’-hydroxywogonin, and hispidulin) of *Scutellaria barbata* and control ligand (resveratrol). (DOCX)

S1 Fig Overlap of breast cancer-associated genes and compound-targeted proteins. The Venn diagram illustrates the unique and shared targets between a curated set of BC-related genes and the combined protein targets of the three candidate flavonoids apigenin, 4′-hydroxywogonin, and hispidulin (39 targets). The 39 unique targets (for compound) were used as the seed proteins for constructing the Protein-Protein Interaction (PPI) network and subsequent analysis.
(DOCX)

S2 Fig Multi-algorithm ranking of hub proteins from the protein–protein interaction (PPI) network. This heatmap visualizes the normalized ranking scores (from 0.0 to 9.0, with 9.0 indicating the highest rank) of the top candidate hub proteins as identified by nine different topological algorithms of the CytoHubba plugin in Cytoscape. Proteins are arranged on the y-axis, while the CytoHubba algorithms scores are displayed on the x-axis. The algorithms include local connectivity-based measures (Degree, Maximum Neighborhood Component (MNC), and Maximal Clique Centrality (MCC) and global centrality-based measures (Betweenness, Closeness, Edge Percolated Component (EPC), BottleNeck, Stress, and Radiality). Key proteins consistently achieving high normalized scores across multiple algorithms such as NFKB1, ESR1, AKT1, TP53, EGFR, and IL6, are identified as high-confidence network hubs, underscoring their central regulatory roles in the flavonoid-targeted breast cancer-associated protein network.
(DOCX)

S3 Fig Pharmacophore modeling and interaction analysis of four lead compounds including (A) apigenin, (B) hispidulin, (C) 4’-hydroxywogonin and (D) resveratrol, highlighting hydrogen bond donors (HBD), acceptors (HBA), and hydrophobic regions. The 3D pharmacophore models (left) and two-dimensional (2D) interaction diagrams (right) illustrate favorable alignment of pharmacophoric features with critical residues within the binding pocket of AKT1.
(DOCX)

S4 Fig Visualization of protein-ligand interactions between the AKT1 protein and ligands (A) apigenin, (B) 4’-hydroxywogonin, (C) hispidulin, and (D) resveratrol (control). (DOCX)

S5 Fig Represented the ligand-protein contact analysis between the AKT1 protein and ligands (A) apigenin, (B) 4’-hydroxywogonin, (C) hispidulin, and (D) resveratrol (control). (DOCX)

S1 File Drug-likeness and toxicity (ADMET) analysis of all phytocompounds from *Scutellaria barbata.* (XLSX)

S2 File Collection and filtering of target genes associated with each ADMET-screened phytochemical. (XLSX)

S3 File List of 387 common genes shared between breast cancer–associated genes and phytochemical-related targets. (XLSX)

S4 File Gene Ontology (GO) enrichment results obtained using the DAVID database. (XLSX)

S5 File Kyoto Encyclopedia of Genes and Genomes (KEGG) pathway enrichment analysis performed using the DAVID database. (XLSX)

S6 File Hub genes identified using 12 topological algorithms of the CytoHubba plugin. (XLSX)
