# Supplementary material for: In silico screening and molecular analyses identify apigenin from Scutellaria barbata as a potent AKT1 inhibitor in breast cancer
Source: PLoS One. 2026 Jun 25;21(6):e0338874. doi: 10.1371/journal.pone.0338874 (PMC13298910; doi:10.1371/journal.pone.0338874)
Supplement: S1 Table — (DOCX) [file pone.0338874.s001.docx]

**S1 Table.** Toxicological properties of bioactive compounds (e.g., apigenin, 4'-hydroxywogonin, and hispidulin) of *Scutellaria barbata*.

| Classification | Target | Apigenin | | 4'-Hydroxywogonin | | Hispidulin | |
| --- | --- | --- | --- | --- | --- | --- | --- |
|  |  | Prediction | Probability | Prediction | Probability | Prediction | Probability |
| Organ toxicity | Hepatotoxicity | Inactive | 0.68 | Inactive | 0.72 | Inactive | 0.72 |
| Organ toxicity | Neurotoxicity | Inactive | 0.86 | Inactive | 0.88 | Inactive | 0.88 |
| Toxicity end points | Carcinogenicity | Inactive | 0.62 | Inactive | 0.68 | Inactive | 0.68 |
| Toxicity end points | Immunotoxicity | Inactive | 0.99 | Inactive | 0.70 | Inactive | 0.72 |
| Toxicity end points | Mutagenicity | Inactive | 0.57 | Inactive | 0.94 | Inactive | 0.94 |
| Toxicityend points | Cytotoxicity | Inactive | 0.87 | Inactive | 0.95 | Inactive | 0.95 |
| Toxicity Class | - | Class IV | - | Class V | - | ClassV | - |
| LD₅₀ (mg/kg) | - | 2500mg/kg | - | 3919mg/kg | - | 4000mg/kg | - |
