# Supplementary material for: In silico screening and molecular analyses identify apigenin from Scutellaria barbata as a potent AKT1 inhibitor in breast cancer
Source: PLoS One. 2026 Jun 25;21(6):e0338874. doi: 10.1371/journal.pone.0338874 (PMC13298910; doi:10.1371/journal.pone.0338874)
Supplement: S2 Table — (DOCX) [file pone.0338874.s002.docx]

**S2 Table.** Physicochemical, drug likeness and pharmacokinetics properties of bioactive compounds (e.g., apigenin, 4'-hydroxywogonin, and hispidulin) of *Scutellaria barbata*.

| Physicochemical properties | Parameter | Apigenin | 4'-Hydroxywogonin | Hispidulin |
| --- | --- | --- | --- | --- |
|  | Molecular weight | 270.24 g/mol | 300.26 g/mol | 300.26 g/mol |
|  | No. of H-bond acceptors | 5 | 6 | 6 |
|  | No. of H-bond donors | 3 | 3 | 3 |
|  | Molar Refractivity | 73.99 | 80.48 | 80.48 |
| Lipophilicity | Consensus Log Po/w | 2.11 | 2.12 | 2.12 |
| Water Solubility | Log S (ESOL) | -3.94 | -3.99 | -3.99 |
|  | Solubility class | Soluble | Soluble | Soluble |
| Drug-likeness | Lipinski | Yes (0 violation) | Yes (0 violation) | Yes (0 violation) |
|  | Ghose | Yes (0 violation) | Yes (0 violation) | Yes (0 violation) |
|  | Veber | Yes (0 violation) | Yes (0 violation) | Yes (0 violation) |
|  | Egan | Yes (0 violation) | Yes (0 violation) | Yes (0 violation) |
|  | Muegge | Yes (0 violation) | Yes (0 violation) | Yes (0 violation) |
|  | Bioavailability Score | 0.55 | 0.55 | 0.55 |
| Medicinal Chemistry | PAINs | 0 alert | 0 alert | 0 alert |
| Absorption | Caco2 permeability | 1.007 | -0.041 | -0.045 |
|  | Intestinal absorption (human) | 93.25 | 87.473 | 84.654 |
|  | Skin Permeability | -2.735 | -2.735 | -2.735 |
|  | P-glycoprotein substrate | Yes | Yes | Yes |
|  | P-glycoprotein I inhibitor | No | No | No |
|  | P-glycoprotein II inhibitor | No | No | No |
| Distribution | VDss (human) | 0.822 | 0.245 | 0.37 |
|  | Fraction unbound (human) | 0.147 | 0.097 | 0.085 |
|  | BBB permeability | -0.734 | -1.108 | -1.12 |
|  | CNS permeability | -2.061 | -2.359 | -2.389 |
| Metabolism | CYP2D6 substrate | No | No | No |
|  | CYP3A4 substrate | No | Yes | Yes |
|  | CYP1A2 inhibitior | Yes | Yes | Yes |
|  | CYP2C19 inhibitior | Yes | Yes | Yes |
|  | CYP2C9 inhibitior | No | Yes | Yes |
|  | CYP2D6 inhibitior | No | No | No |
|  | CYP3A4 inhibitior | No | No | No |
| Excretion | Total Clearance | 0.566 | 0.513 | 0.531 |
|  | Renal OCT2 substrate | No | No | No |
