# Supplementary material for: In silico screening and molecular analyses identify apigenin from Scutellaria barbata as a potent AKT1 inhibitor in breast cancer
Source: PLoS One. 2026 Jun 25;21(6):e0338874. doi: 10.1371/journal.pone.0338874 (PMC13298910; doi:10.1371/journal.pone.0338874)
Supplement: S3 Table — (DOCX) [file pone.0338874.s003.docx]

**S3 Table.** Topological characteristics of identified three hub proteins.

| Algorithm | AKT1 | IL6 | TNF |
| --- | --- | --- | --- |
| Degree | 238 | 233 | 232 |
| MNC | 237 | 233 | 232 |
| MCC | 7.38E+48 | 7.38E+48 | 7.38E+48 |
| Betweenness | 5843.694309 | 3660.859313 | 3695.415743 |
| Closeness | 5843.694309 | 305.9166667 | 305.25 |
| BottleNeck | 6 | 9 | 21 |
| ClusteringCoefficient | - | - | - |
| DMNC | - | - | - |
| EcCentricity | - | - | - |
| EPC | 16.983 | 16.507 | 15.399 |
| Radiality | 5.627296588 | 5.611548556 | 5.606299213 |
| Stress | 113228 | 102248 | 98174 |

*Here: MNC; maximum neighborhood component, MCC; maximal clique centrality, DMNC; density of maximum neighborhood component and EPC; edge percolated component.*
