# Supplementary material for: In silico screening and molecular analyses identify apigenin from Scutellaria barbata as a potent AKT1 inhibitor in breast cancer
Source: PLoS One. 2026 Jun 25;21(6):e0338874. doi: 10.1371/journal.pone.0338874 (PMC13298910; doi:10.1371/journal.pone.0338874)
Supplement: S4 Table — (DOCX) [file pone.0338874.s004.docx]

**S5 Table.** Docking score and non-bond interactions of apigenin, 4'-hydroxywogonin, and hispidulin against selected IL6 and TNF hub proteins of breast cancer.

| Proteins  (PDB ID) | Compound (CID) | PubChem  CID | Docking Score  (kcal/mol) | Hydrogen bond  interaction | Hydrophobic bond interaction |
| --- | --- | --- | --- | --- | --- |
|  |  |  |  | Residues involved | Residues involved |
| IL6  (1 ALU) | Apigenin | 5280443 | -5.9 | ASN144, PRO139, GLN127, LYS120, and PRO141 | LEU92, ILE123, PRO139, and PRO141 |
|  | 4'-hydroxywogonin | 5322078 | -5.8 | LYS120, ASN144, GLN116, PRO139, and LYS120 | LEU92 |
|  | Hispidulin | 5281628 | -5.8 | GLN127, ASN144, PRO139, and LYS120 | LEU92, ILE123, PRO139, and PRO141 |
|  | Tartaric acid (control) | 444305 | -3.9 | ASN144, and GLU99 |  |
| TNF  (7JRA) | Apigenin | 5280443 | -6.5 | GLN97, ALA221, and PHE220 | PHE220 |
|  | 4'-hydroxywogonin | 5322078 | -6.2 | LYS141, PRO96, and PHE220 | PHE220, ALA221, and LYS141 |
|  | Hispidulin | 5281628 | -6.4 | PHE220, ALA98, and ASP216 | PHE220 |
|  | Lenalidomide (control) | 216326 | -6 | PRO215, LEU218, GLN97, and GLY100 | PHE220 |
