# Supplementary material for: In silico screening and molecular analyses identify apigenin from Scutellaria barbata as a potent AKT1 inhibitor in breast cancer
Source: PLoS One. 2026 Jun 25;21(6):e0338874. doi: 10.1371/journal.pone.0338874 (PMC13298910; doi:10.1371/journal.pone.0338874)
Supplement: S5 Table — (DOCX) [file pone.0338874.s005.docx]

**S6 Table.** Energy of highest occupied molecular orbitals (HOMOs), lowest unoccupied molecular orbitals (LUMOs), gaps, hardness, and softness of phytocompounds (e.g., apigenin, 4'-hydroxywogonin, and hispidulin) of *Scutellaria barbata* and control ligand (resveratrol).

| Phytocompounds | *ɛ*HOMO | *ɛ*LUMO | Gap | (*η*) (Hardness, Gap/2) | *S* (Softness, 1/Hardness) |
| --- | --- | --- | --- | --- | --- |
| Resveratrol (control) | -0.19275 | -0.04437 | 0.14838 | 0.07419 | 13.47 |
| Apigenin | -0.21663 | -0.06386 | 0.15277 | 0.07638 | 13.09 |
| 4'-Hydroxywogonin | -0.21361 | -0.06387 | 0.14974 | 0.07487 | 13.35 |
| Hispidulin | -0.20776 | -0.06294 | 0.14482 | 0.07241 | 13.81 |
