# Supplementary material for: In silico screening and molecular analyses identify apigenin from Scutellaria barbata as a potent AKT1 inhibitor in breast cancer
Source: PLoS One. 2026 Jun 25;21(6):e0338874. doi: 10.1371/journal.pone.0338874 (PMC13298910; doi:10.1371/journal.pone.0338874)
Supplement: S1 Fig — The Venn diagram illustrates the unique and shared targets between a curated set of BC-related genes and the combined protein targets of the three candidate flavonoids apigenin, 4′-hydroxywogonin, and hispidulin (39 targets). The 39 unique targets (for compound) were used as the seed proteins for constructing the Protein-Protein Interaction (PPI) network and subsequent analysis. (DOCX) [file pone.0338874.s006.docx]

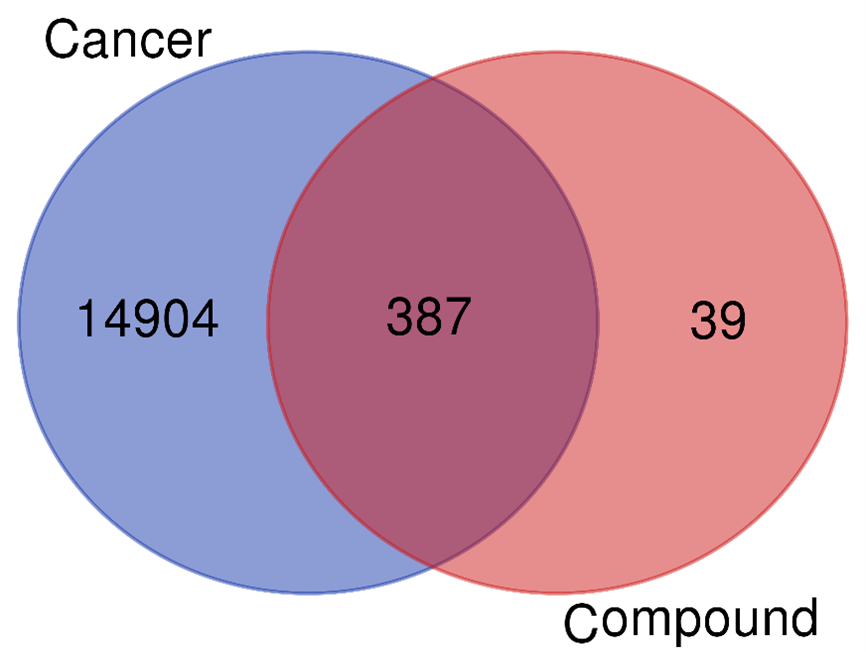


**S1 Fig.** Overlap of breast cancer-associated genes and compound-targeted proteins. The Venn diagram illustrates the unique and shared targets between a curated set of BC-related genes and the combined protein targets of the three candidate flavonoids apigenin, 4′-hydroxywogonin, and hispidulin (39 targets). The 39 unique targets (for compound) were used as the seed proteins for constructing the Protein-Protein Interaction (PPI) network and subsequent analysis.
