# Supplementary material for: In silico screening and molecular analyses identify apigenin from Scutellaria barbata as a potent AKT1 inhibitor in breast cancer
Source: PLoS One. 2026 Jun 25;21(6):e0338874. doi: 10.1371/journal.pone.0338874 (PMC13298910; doi:10.1371/journal.pone.0338874)
Supplement: S2 Fig — This heatmap visualizes the normalized ranking scores (from 0.0 to 9.0, with 9.0 indicating the highest rank) of the top candidate hub proteins as identified by nine different topological algorithms of the CytoHubba plugin in Cytoscape. Proteins are arranged on the y-axis, while the CytoHubba algorithms scores are displayed on the x-axis. The algorithms include local connectivity-based measures (Degree, Maximum Neighborhood Component (MNC), and Maximal Clique Centrality (MCC) and global centrality-based measures (Betweenness, Closeness, Edge Percolated Component (EPC), BottleNeck, Stress, and Radiality). Key proteins consistently achieving high normalized scores across multiple algorithms such as NFKB1, ESR1, AKT1, TP53, EGFR, and IL6, are identified as high-confidence network hubs, underscoring their central regulatory roles in the flavonoid-targeted breast cancer-associated protein network. (DOCX) [file pone.0338874.s007.docx]

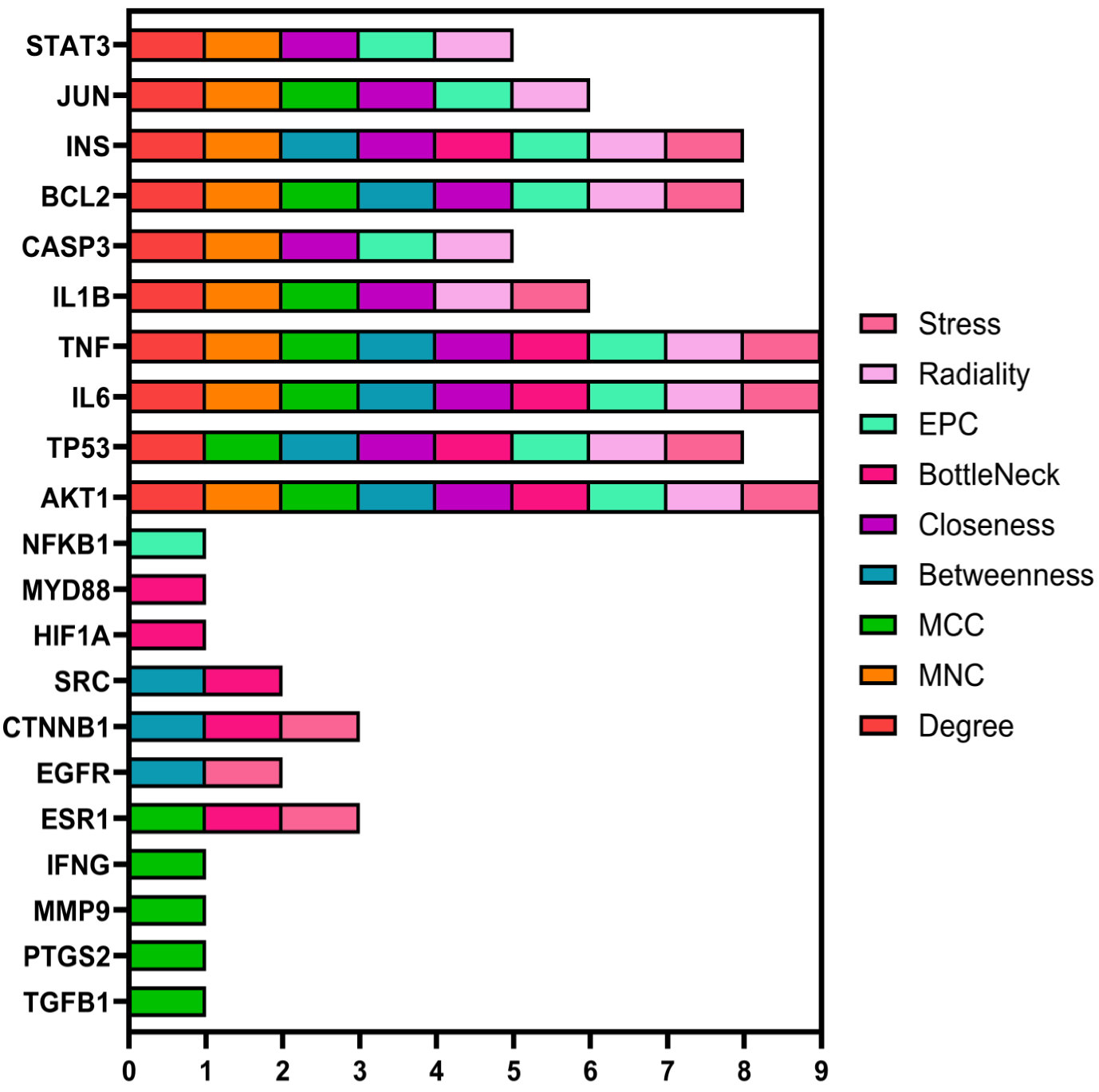


**S2 Fig.** Multi-algorithm ranking of hub proteins from the protein–protein interaction (PPI) network. This heatmap visualizes the normalized ranking scores (from 0.0 to 9.0, with 9.0 indicating the highest rank) of the top candidate hub proteins as identified by nine different topological algorithms of the CytoHubba plugin in Cytoscape. Proteins are arranged on the y-axis, while the CytoHubba algorithms scores are displayed on the x-axis. The algorithms include local connectivity-based measures (Degree, Maximum Neighborhood Component (MNC), and Maximal Clique Centrality (MCC) and global centrality-based measures (Betweenness, Closeness, Edge Percolated Component (EPC), BottleNeck, Stress, and Radiality). Key proteins consistently achieving high normalized scores across multiple algorithms such as NFKB1, ESR1, AKT1, TP53, EGFR, and IL6, are identified as high-confidence network hubs, underscoring their central regulatory roles in the flavonoid-targeted breast cancer-associated protein network.
