# Supplementary material for: In silico screening and molecular analyses identify apigenin from Scutellaria barbata as a potent AKT1 inhibitor in breast cancer
Source: PLoS One. 2026 Jun 25;21(6):e0338874. doi: 10.1371/journal.pone.0338874 (PMC13298910; doi:10.1371/journal.pone.0338874)
Supplement: S3 Fig — The 3D pharmacophore models (left) and two-dimensional (2D) interaction diagrams (right) illustrate favorable alignment of pharmacophoric features with critical residues within the binding pocket of AKT1. (DOCX) [file pone.0338874.s008.docx]

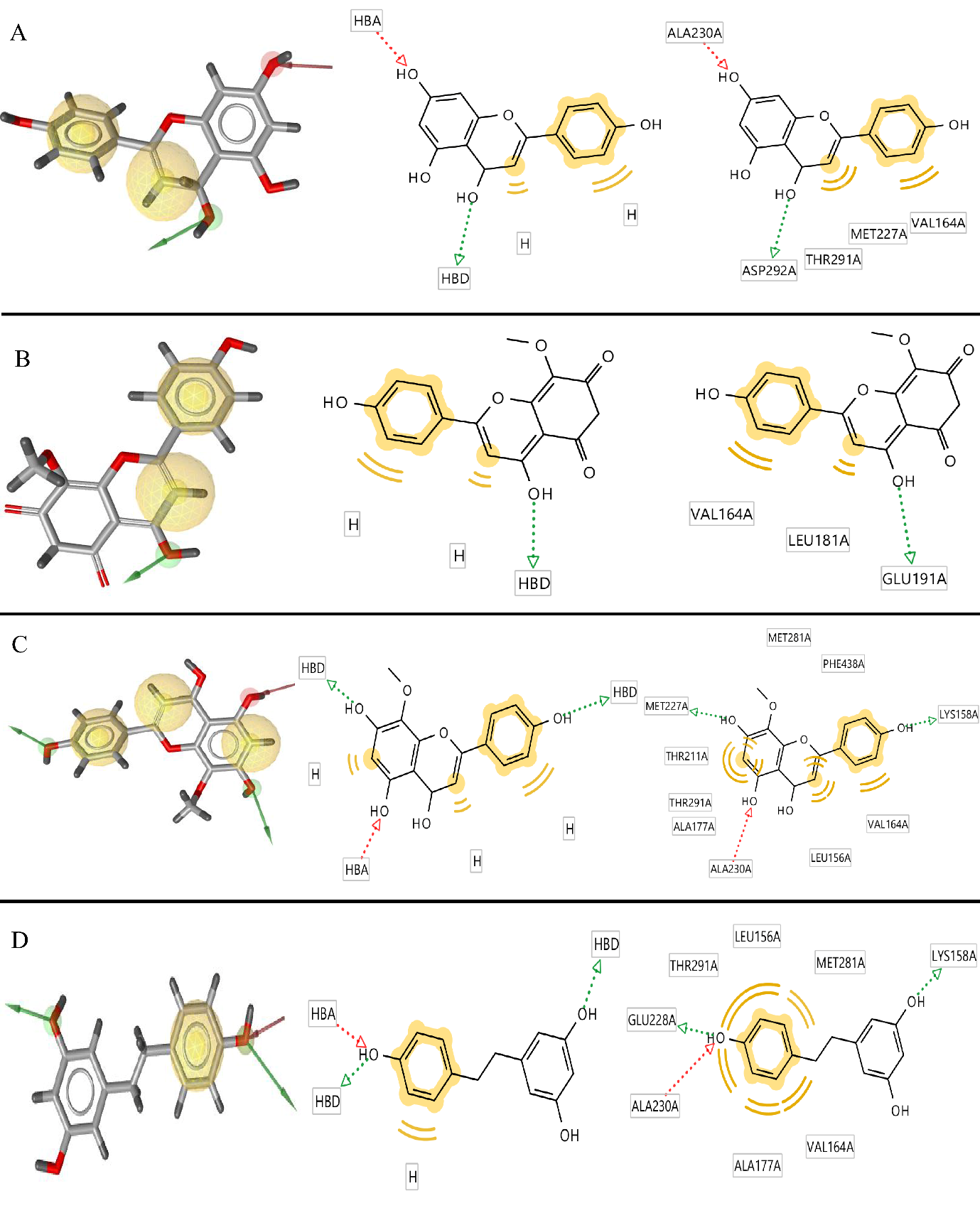


**S3 Fig.** Pharmacophore modeling and interaction analysis of four lead compounds including (A) apigenin, (B) hispidulin, (C) 4'-hydroxywogonin and (D) resveratrol, highlighting hydrogen bond donors (HBD), acceptors (HBA), and hydrophobic regions. The 3D pharmacophore models (left) and two-dimensional (2D) interaction diagrams (right) illustrate favorable alignment of pharmacophoric features with critical residues within the binding pocket of AKT1.
