# Supplementary material for: In silico screening and molecular analyses identify apigenin from Scutellaria barbata as a potent AKT1 inhibitor in breast cancer
Source: PLoS One. 2026 Jun 25;21(6):e0338874. doi: 10.1371/journal.pone.0338874 (PMC13298910; doi:10.1371/journal.pone.0338874)
Supplement: S4 Fig — (DOCX) [file pone.0338874.s009.docx]

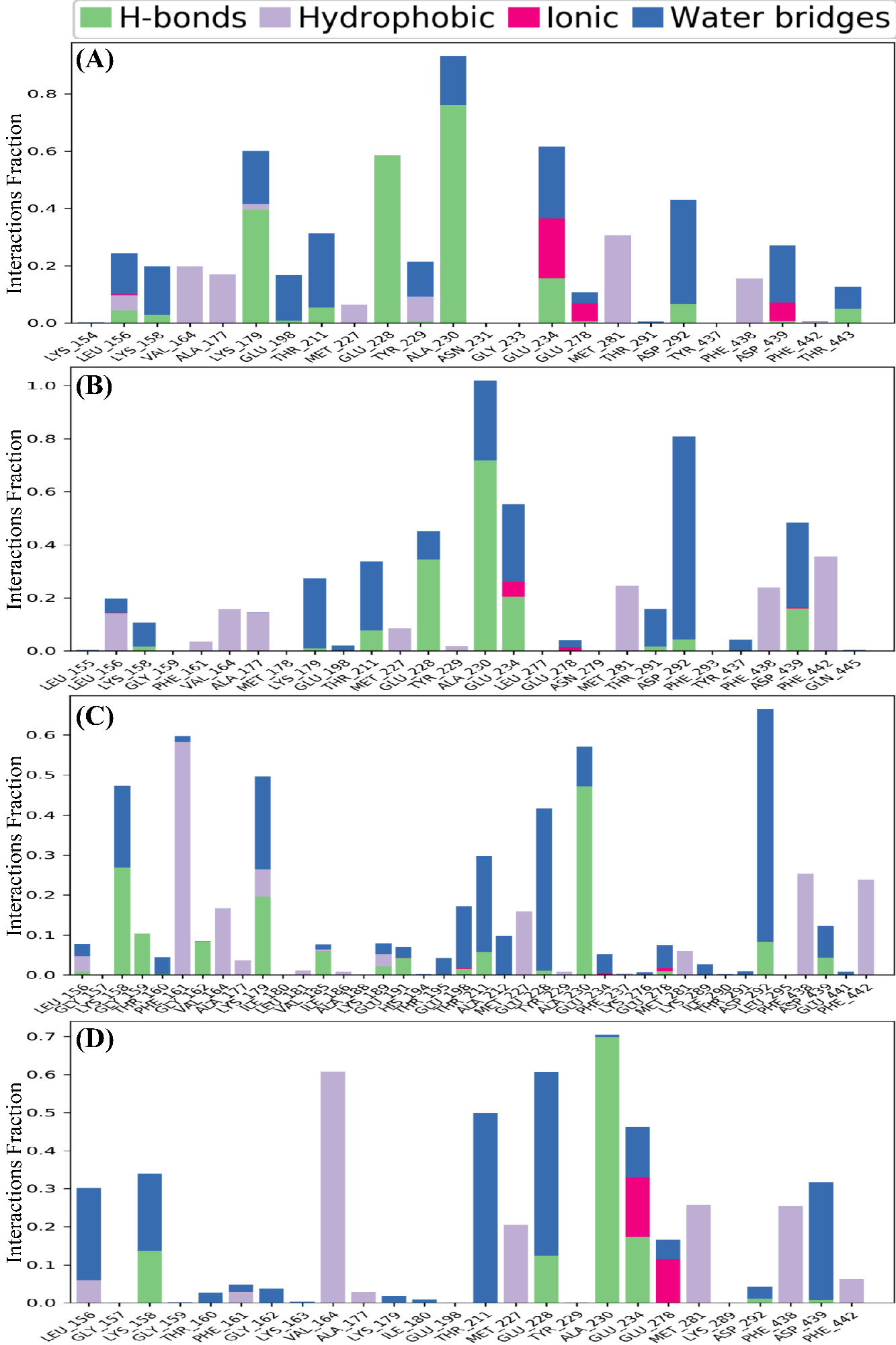


**S4 Fig.** Visualization of protein-ligand interactions between the AKT1 protein and ligands (A) apigenin, (B) 4'-hydroxywogonin, (C) hispidulin, and (D) resveratrol (control).
