# Supplementary material for: In silico screening and molecular analyses identify apigenin from Scutellaria barbata as a potent AKT1 inhibitor in breast cancer
Source: PLoS One. 2026 Jun 25;21(6):e0338874. doi: 10.1371/journal.pone.0338874 (PMC13298910; doi:10.1371/journal.pone.0338874)
Supplement: Attachment — Submitted filename: Responses to Reviewers.pdf [file pone.0338874.s018.pdf]

Response to Reviewer #1 Comments

Comment 1: Overinterpretation of computational findings (Lines ~38–41, 546–612).
The manuscript frequently implies definitive biological activity based solely on computational evidence. PLOS ONE requires that conclusions be supported strictly by the presented data. Statements such as "apigenin is a promising multi-target flavonoid for combating breast cancer" (Line 38) or references to "therapeutic potential" should be tempered.

Our Response: We thank the reviewer for this valuable and constructive comment. We agree that computational analyses alone do not establish definitive biological activity or therapeutic efficacy. In accordance with PLOS ONE's guidelines, we have revised the manuscript to ensure that all conclusions are strictly supported by the presented in silico data.
Specifically, the statements in Lines 38-41 and (546-612) have been rewritten to avoid implications of confirmed biological or therapeutic effects. Language suggesting that apigenin "combats breast cancer" or possesses direct "therapeutic potential" has been replaced with more cautious phrasing that emphasizes computational predictions and the hypothesis-generating nature of the study. Please refer to the updated manuscript.

Comment 2: Lack of clarity in methodology and insufficient reproducibility in several sections Although the Methods section is detailed overall, certain areas require clarification to ensure reproducibility:
a. Dataset reproducibility (Lines 289–307, 331–340)
• The authors list total numbers of genes/targets, but the raw gene lists are not provided in supplementary files.
• It is unclear how duplicate genes were removed (based on gene symbol, UniProt ID, Entrez ID?).

Our Response: We thank the reviewer for highlighting these important reproducibility concerns. To address this issue, we have taken the following steps.
First, we have updated the manuscript to improve methodological transparency and ensure full reproducibility of the dataset construction process. The complete raw and processed data corresponding to Lines 289–307 have now been provided as **Supplementary Files S1-S7**. These include: (i) all phytochemical information collected from multiple databases; (ii) oral bioavailability and drug-likeness scores; and (iii) toxicity prediction results.
Secondly, for "lines 331–340" we have updated the result section where the removal process of duplicate genes (based on gene symbol) is clearly mentioned and the complete gene list is provided as Supplementary files. Each list includes standardized official gene symbols and corresponding identifiers to facilitate reproducibility. Please refer to the updated manuscript.

b. Docking methodology (Lines 213–227)
• Grid box dimensions are listed, but grid box centers (x,y,z) are not fully specified for all proteins.

Our Response: We thank the reviewer for pointing out this important reproducibility issue. We agree that specifying both grid box dimensions and center coordinates is necessary for accurate reproduction of docking results. To address this concern, we have now updated the Methods section (Lines 213–227) where we provided the information of the complete grid box center coordinates (x, y, z) along with grid dimensions for all target proteins. Please refer to **Lines 230-235** in the updated manuscript.

• Justification for control drugs (resveratrol, tartaric acid, etc.) should be strengthened, as some are not standard inhibitors of the target proteins.

Response: We thank the reviewer for this insightful comment. The control compounds lenalidomide was used as a reference compound due to its reported in vivo antitumor activity mediated by immunomodulatory effects, including suppression of TNF-α [47, 48], whereas resveratrol was included as a biologically relevant reference compound based on previously published in silico and experimental evidence demonstrating its direct interaction with AKT and its modulatory role via NQO2 [49, 50]. In contrast, tartaric acid was selected based on previously published in silico studies and its presence in IL-6 crystal structures [51, 52]. This has been briefly discussed in the Methods section. Please refer to **Lines 209-215** in the updated manuscript.

c. MD simulations (Lines 241–258)
• The RMSD and RMSF analyses are descriptive, but statistical summaries (mean ± SD for stable intervals) would help interpretation.
• Figures should show ligand-only RMSD, not only protein–ligand complex RMSD.

Response: We thank the reviewer for this helpful suggestion. To improve the interpretability of the molecular dynamics analyses, we have now calculated and reported the mean ± standard deviation of RMSD and RMSF values in the result and discussion sections. In addition, ligand-only RMSD plots have now been generated and included in the revised figures to complement the protein-ligand complex RMSD analyses. These additions provide a clearer assessment of ligand stability within the binding pocket during the simulation. Please refer to **Lines 475-480** and **Lines 483-486** in the updated manuscript.

Comment 3: Network pharmacology interpretation requires refinement (Lines 344–384) The enrichment results include many infection-related pathways (e.g., EBV, hepatitis B/C). These are common artifacts of over-broad gene lists.

Response: We thank the reviewer for this insightful comment. We agree that infection-related pathways may arise from broadly enriched gene sets and may obscure disease-specific interpretation. Following the reviewer's alternative recommendation, we have reorganized and refined the KEGG pathway analysis to focus exclusively on cancer-related and signal-transduction pathways relevant to breast cancer. Infection-related pathways were excluded from the revised analysis, and the corresponding KEGG enrichment figure has been updated to emphasize biologically meaningful pathways involved in tumorigenesis, cell proliferation, apoptosis, and key signaling cascades. These revisions have been incorporated into the Results and Discussion sections to improve mechanistic clarity and disease relevance. Please refer to **Lines 416-424** and **Lines 483-486** in the updated manuscript.

Comment 4: Hub protein selection requires more rigorous justification (Lines 386–401)

While the use of five CytoHubba algorithms is commendable, the integration strategy is unclear.
Specific gaps:
• The manuscript does not explain how the four proteins were chosen when multiple algorithms produced larger sets.
• No statistical robustness measure is provided.

Our Response: We thank the reviewer for this valuable comment regarding hub protein selection. We would like to clarify that in the earlier version of the manuscript; four hub proteins were

identified based on a limited subset of CytoHubba algorithms. Following the suggestions from the reviewers, we expanded the analysis to include all twelve CytoHubba algorithms (MCC, DMNC, MNC, Degree, EPC, BottleNeck, EcCentricity, Closeness, Radiality, Betweenness, Stress, and Clustering Coefficient) to improve methodological rigor and robustness. To integrate the results objectively, we implemented a consensus-based selection criterion, whereby proteins appearing in ≥9 out of 12 algorithm rankings were considered robust hub proteins. After applying this strict and more comprehensive integration strategy, only three proteins (AKT1, IL6, and TNF) consistently satisfied the consensus threshold. As a result, the previously identified protein (BCL2) did not meet the updated robustness criteria and was therefore excluded. This refinement reflects a more stringent, unbiased, and statistically robust hub selection process, rather than a contradiction of the earlier findings. Relevant methodological details and topological parameters have now been clearly described in the revised manuscript. Please refer to **Lines 182-192** in the updated manuscript.

5. English expression, clarity, and grammar need improvement (Lines 48–120, 546–612)

The manuscript reads clearly in many sections, but substantial grammatical and stylistic issues remain

Our Response: We thank the reviewer for highlighting the need to improve English expression, clarity, and grammar. We confirm that all suggested corrections have been carefully addressed. The manuscript has been thoroughly revised to improve grammatical accuracy, sentence structure, clarity, and overall readability. Stylistic inconsistencies and ambiguous phrasing have been corrected to ensure a clear and professional scientific presentation. We believe these revisions have substantially improved the quality and coherence of the manuscript.

Minor Comments

Comment 1: Figures

o Some figure legends are incomplete (particularly Figs. 6 and 7).

Response: We thank the reviewer for this comment. The figure legends for **Figs. 6 and 7** have been revised to provide complete and self-contained descriptions. The updated legends now clearly explain all panels, define ligand labels (L1–L4), specify simulation details, and describe the meaning of the violin plots and calculated parameters.

o Complex figures (e.g., S3–S4) require higher resolution.

Response: We thank the reviewer for this suggestion. Figures S3 and S4 have been updated and provided in higher resolution (600 dpi) to ensure clarity of all details, including text labels and data points.

Comment 2: Units and formatting

o In some cases, spacing between numbers and units is inconsistent (e.g., “100 ns”, “0.14838 a.u.”).

o Please ensure uniform use of italic formatting for variables such as p, η, S, etc.

Response: We thank the reviewer for pointing this out. We have carefully reviewed the manuscript and ensured consistent spacing between numbers and units throughout (e.g., "100 ns", "0.14838 a.u."). Additionally, all variables (p, η, S, etc.) are now formatted in italics consistently across the text, figures, and tables.

Comment 3: Toxicity classification (Lines 289–307)

o The authors mention "class 4 and 5" toxicity but do not provide the criteria for these classes.

o Please reference OECD or ProTox categories explicitly.

Response: We thank the reviewer for this important comment. The toxicity classes have now been explicitly defined in result section based on ProTox-3 predictions following globally harmonized system of classification of labelling of chemicals (GHS) criteria. Specifically, Class IV (harmful if swallowed; LD_{50} 300 < LD50 ≤ 2000 mg/kg) and Class V (may be harmful if swallowed; LD_{50} 2000 < LD50 ≤ 5000 mg/kg) were assigned according to the predicted LD_{50} values. Please refer to **Lines 321-326** in the updated manuscript.

Comment 4: References

o Some citations are outdated given the rapidly evolving field of computational oncology.

o Please update key background references (e.g., network pharmacology, MD in drug discovery).

Response: We thank the reviewer for this valuable comment. We have updated the key references in the manuscript to include more recent studies in computational oncology, network pharmacology, and MD-based drug discovery. Outdated citations have been replaced with current, high-impact literature to better reflect the state of the field. Please refer to the updated manuscript.

Response to Reviewer ##2 Comments

Comment 1: What does it meant by "compounds lacking SMILES identifiers"? if the compounds have no smiles, so they were removed? Please explain.

Our Response: We thank the reviewer for this comment. Compounds lacking SMILES identifiers are those for which a standardized, machine-readable representation of their chemical structure was not available. Since all in silico prediction tools require SMILES as input to accurately calculate physicochemical properties, toxicity, and other molecular features, these compounds could not be processed reliably. Therefore, to ensure the integrity and reproducibility of our computational analyses, all compounds without valid SMILES scores were systematically excluded from further analysis. We have updated the draft and additional file has been uploaded as supplementary file. Please refer to the updated submission.

Comment 2: Line 163 and onward, it seems confusion here, the authors try PPI, which is normally among the overlapping genes between the disease and compounds, then the authors add here compounds, please recheck this description.

Our Response: We thank the reviewer for this observation. We agree that the previous description could be misleading. We have updated the manuscript to clarify that the PPI network was constructed using only

the target proteins of apigenin, 4′-hydroxywogonin, and hispidulin that overlap with breast cancer-related genes, ensuring that only relevant proteins were included. Please refer to the updated manuscript.

Comment 3: The binding scores are reported in the discussion, but it doesn't critically compare these compounds with established breast cancer drugs or known P13K/AKT inhibitors, further references are needed to support the claim.

Our Response: We thank the reviewer for this valuable suggestion. To provide a proper comparative context, we have included well-characterized AKT inhibitors, capivasertib and ipatasertib, as reference compounds in the discussion. Capivasertib has been reported to bind strongly to AKT1 (−9.5 kcal/mol) and AKT2 (−8.4 kcal/mol), while ipatasertib binds favorably to AKT isoforms, particularly AKT2 (−7.2 kcal/mol). The AKT1 binding affinity observed for hispidulin (−8.1 kcal/mol) and apigenin (−7.7 kcal/mol) in our study falls within a comparable range, supporting the plausibility of AKT-targeted interactions. We have updated the discussion section accordingly and included these references to strengthen the interpretation of our docking results. Please refer to the updated manuscript.

Comment 4: The GO and KEGG pathway enrichment results largely repeate well-known cancer pathways, displaying limited novel mechanistic understanding specific to breast cancer subtypes.

Our Response: We thank the reviewer for this valuable comment. We acknowledge that many enriched GO terms and KEGG pathways correspond to well-established cancer-related processes, reflecting the biological relevance of the compound targets in breast cancer. While some pathways are widely recognized, our analysis provides a systematic integration of compound-target-disease associations, highlighting how apigenin, 4′-hydroxywogonin, and hispidulin may collectively modulate these pathways. Furthermore, the enrichment results serve as a foundation for future investigations, particularly for exploring their effects in specific breast cancer subtypes, which can be validated experimentally. Please refer to the updated manuscript.

Comment 5: The introduction should be further strengthened by the addition of importance of network pharmacology and molecular docking, the author can take information form these references or any other relevant one (PMID: 40764661, PMID: 41096765)

Our Response: We thank the reviewer for this constructive suggestion. The introduction has been revised to highlight the significance of network pharmacology in identifying multi-target interactions and elucidating potential mechanisms of phytochemicals in complex disease pathways. Additionally, the importance of molecular docking as a computational tool to predict ligand–protein interactions and prioritize potential bioactive compounds has been emphasized. Relevant information from the suggested references (PMID: 40764661, PMID: 41096765) has been incorporated to strengthen the rationale for our computational approach. Please refer to **Lines 84-96** in the updated manuscript.

Comment 6: Please provide all the relevant supporting information regarding network pharmacology, it should be the data obtained from different databases.

Our Response: We thank the reviewer for this suggestion. All relevant data supporting the network pharmacology analysis, including compound related-target, disease-related genes, overlapping targets/common gene, and PPI network details obtained from STRING database, have now been compiled and provided in Supplementary file. Please refer to the updated manuscript.

<u>Response to Reviewer #3 Comments</u>

Major Comments

Comment 1: Network pharmacology and KEGG analyses identify largely generic cancer and inflammatory pathways. Greater emphasis on breast cancer–specific relevance and mechanistic prioritization is needed.

Our Response: We thank the reviewer for this valuable comment. We agree that an overrepresentation of generic cancer and inflammatory pathways may limit disease-specific interpretation. To address this, we have revised the KEGG pathway analysis and updated the corresponding figure to emphasize breast cancer–specific and signal-transduction–related pathways, prioritizing key mechanisms involved in breast cancer progression. These revisions improve mechanistic focus and disease relevance and have been incorporated into the Results and Discussion sections. Please refer to the updated manuscript.

Comment 2: Molecular dynamics simulations were carried out only for AKT1, even though several key target proteins were identified. Authors are requested to perform dynamics for other targets proteins as well.

Our Response: We thank the reviewer for this suggestion. Although three hub genes (AKT1, IL6 and TNF) were identified from the PPI network, AKT1 exhibited the most favorable docking scores with the selected ligands compared to the other hub proteins. Therefore, AKT1 was prioritized as the representative target for molecular dynamics simulations. Due to computational scope and resource limitations, extending molecular dynamics simulations to all hub proteins was beyond the scope of the present study. This limitation has now been clearly stated in the revised manuscript (**Lines 657-663**).

Comment 3: Target prioritization is insufficiently justified. Although multiple hub proteins were identified, the rationale for emphasizing AKT1 and apigenin in particular—is unclear, especially since hispidulin shows stronger binding in docking and MM-GBSA analyses. Thus, authors are requested to give information on this point as well.

Our Response: We thank the reviewer for this comment. Among the identified hub proteins, AKT1 was selected as the primary target because it exhibited the most favorable docking scores with the screened ligands compared to other hub proteins. Although hispidulin showed stronger binding affinity toward AKT1 in docking and MM-GBSA analyses, ligand prioritization was based on dynamics stability rather than static binding energy alone.

During the 100 ns molecular dynamics simulations, the apigenin–AKT1 complex demonstrated superior stability, reflected by lower RMSD and RMSF fluctuations, stable radius of gyration, and consistent SASA profiles compared with hispidulin and the control compound. Since docking and MM-GBSA mainly capture static interactions and do not fully reflect dynamic behavior, apigenin was prioritized based on its more stable and equilibrated binding mode. These points have now been clarified in the Abstract and Results sections. Please refer to the updated manuscript.

Comment 4: Compound filtering based on ADME thresholds (OB, DL) may bias selection and exclude potentially active phytochemicals. The authors are requested to give clear justification for their choice of cut-offs.

Our Response: We thank the reviewer for this important comment. The oral bioavailability (OB) and drug-likeness (DL) thresholds used in our study (OB ≥ 30%, DL ≥ 0.18) were selected based on widely accepted standards in network pharmacology research to ensure that the included compounds have sufficient systemic exposure and favorable pharmacokinetic properties. While we acknowledge that applying these thresholds may exclude some potentially active phytochemicals, this approach prioritizes compounds with higher translational potential, improving the reliability and relevance of downstream network pharmacology and molecular docking analyses. The rationale for these cut-offs has now been explicitly stated in the Methods section and additional citation has also been added for this criterion. Please refer to **Lines 127-136** in the updated manuscript.

Comment 5: Entropy was not included in the MM-GBSA calculations; the authors should explain this omission and discuss how it may affect the interpretation of the binding energy results.

Our Response: We thank the reviewer for raising this important point. Entropic contributions (−TΔS) were not included in the MM-GBSA calculations because entropy estimation is computationally expensive and is often associated with large statistical uncertainty, particularly when derived from normal-mode or quasi-harmonic analyses. Several previous studies have demonstrated that MM-GBSA calculations performed without explicit entropy terms can still reliably rank ligands and compare relative binding affinities, provided that a consistent computational protocol is applied across all systems (e.g., Genheden & Ryde, 2015; DOI: https://doi.org/10.1517/17460441.2015.1032936, and Xu L et al., 2013; DOI: https://doi.org/10.1021/jp404160y). Accordingly, the binding energies reported in this study should be interpreted as relative binding free energies rather than absolute values. While omission of the entropic term may lead to an overestimation of absolute binding energies, it does not compromise the comparative evaluation or ranking of the ligands examined. This clarification has now been explicitly stated in the revised manuscript to ensure appropriate interpretation of the MM-GBSA results.

Minor Comments

Comment 1: Abbreviations (e.g., RA, AEs, SAEs, DMARDs) should be defined at first mention in the main text and used consistently thereafter.

Our Response: We thank the reviewer for this comment. All abbreviations have now been defined at their first occurrence in the main text and are used consistently throughout the manuscript.

Comment 2: Minor inconsistencies in referencing style and citation formatting should be corrected.

Our Response: We thank the reviewer for this comment. All references and in-text citations have been carefully revised to follow PLOS ONE's citation style, ensuring consistency throughout the manuscript.

Comment 3: The resolution and figure legend of several figures should be improved for better understanding and readability.

Our Response: We sincerely thank the reviewer for this helpful suggestion. The figures identified as having resolution and legend clarity issues have been carefully revised and updated to improve image quality and enhance the explanatory details in their legends (**especially Figs. 1, 3, 4 and 6**). Figures that were already clear and met the journal's quality standards were therefore left unchanged. All revised figures are included in the updated manuscript.

Comment 4: Minor inconsistencies in referencing style and citation formatting should be corrected to align with the journal's guidelines.

Our Response: We thank the reviewer for this comment. All references and in-text citations have been carefully reviewed and corrected to ensure full compliance with PLOS ONE's referencing and citation guidelines, maintaining consistency throughout the manuscript.
