# Supplementary material for: In silico screening and molecular analyses identify apigenin from Scutellaria barbata as a potent AKT1 inhibitor in breast cancer
Source: PLoS One. 2026 Jun 25;21(6):e0338874. doi: 10.1371/journal.pone.0338874 (PMC13298910; doi:10.1371/journal.pone.0338874)
Supplement: Attachment — Submitted filename: Responses_to_Reviewers_auresp_2.pdf [file pone.0338874.s019.pdf]

<u>Response to Reviewer #1 Comments</u>

Comment 1: This manuscript presents a computational investigation into apigenin as a putative AKT1 inhibitor, contributing to the growing literature on phytochemicals as potential anticancer agents. The study is relevant, clearly motivated, and methodologically coherent; however, several areas require clarification and strengthening before the conclusions can be fully supported.

Our Response: We thank the reviewer for this valuable and constructive comment. We agree that computational analyses alone do not establish definitive biological activity or therapeutic efficacy. Accordingly, we have carefully revised the manuscript to clarify the scope and limitations of the present in-silico study. Specifically, we now emphasize that our findings identify apigenin as a potential AKT1 inhibitor based on molecular docking and simulation analyses, rather than confirming biological potency. We have also moderated the language in the title, abstract, and discussion to avoid overinterpretation and to clearly position our results as a hypothesis-generating framework that may guide future in-vitro and in-vivo validation studies.

Comment 2: Title (Lines 1–2)

• The title is clear and accurately reflects the computational nature of the study.

• Suggestion: Consider adding “in silico” to the title to avoid overinterpretation of biological efficacy.

Our Response: We thank the reviewer for this helpful suggestion. To avoid overinterpretation of biological efficacy and to more clearly reflect the computational nature of the study, we have revised the title to explicitly include the term “in silico.” This modification ensures that the scope and limitations of the work are clearly communicated to readers and aligns the title with the methodology employed in the study. Please refer to the revised title (***In Silico* Screening and Molecular Analyses Identify Apigenin from *Scutellaria barbata* as a Potent AKT1 Inhibitor in Breast Cancer**)

Comment 3: Abstract (Lines 20–45)

• The abstract summarizes the study well.

• Concern: Phrases suggesting therapeutic potency should be tempered to reflect computational prediction rather than experimental validation.

Our Response: We thank the reviewer for this constructive comment. We have carefully revised the abstract to moderate any statements implying therapeutic efficacy and to clearly indicate that our findings are based on in silico predictions rather than experimental validation. All phrases suggesting therapeutic potency have been reworded to reflect computational screening and theoretical potential only. Please refer to the **Abstract** in the updated manuscript.

Comment 4: Introduction (Lines 50–130)

• The background on AKT1 signaling and breast cancer is well articulated.

• Line 95–110: The rationale for selecting *Scutellaria barbata* and apigenin should be more explicitly linked to prior experimental or clinical evidence.

Our Response: We appreciate the reviewer's insightful suggestion. In response, we have revised the Introduction (Lines 95–110) to strengthen the rationale for selecting *Scutellaria barbata* and its bioactive compound apigenin by explicitly linking their selection to prior experimental and reported clinical evidence. Specifically, we have added supporting references describing the documented anticancer activities of *S. barbata* and the inhibitory effects of apigenin on breast cancer–related signaling pathways, including AKT1-associated mechanisms. These additions clarify the biological relevance of the chosen plant and compound and better justify their selection for the present computational investigation. Please refer to **Lines 97-112** in the updated manuscript.

Comment 5: Materials and Methods (Lines 140–260)
• Docking and computational tools are appropriately selected.
• Line 175–190: Protein and ligand preparation steps require more detail to ensure reproducibility.
• Please specify docking validation procedures (e.g., re-docking, RMSD thresholds).

Our Response: We sincerely thank the reviewer for this valuable suggestion. We have expanded the Materials and Methods section (**Lines 191–213**) to provide detailed descriptions of protein and ligand preparation, including protein structure retrieval, removal of water molecules and heteroatoms, addition of polar hydrogens, assignment of charges, and ligand energy minimization and format conversion prior to docking.
In addition, we have now explicitly described the docking validation procedure. The co-crystallized ligand was re-docked into the active site of the target protein, and the predicted pose was compared with the native binding conformation. Docking accuracy was evaluated using root mean square deviation (RMSD), and an RMSD value $\leq$ 2.0 Å was considered acceptable, confirming the reliability of the docking protocol. Please refer to **Lines 191-213** and **Lines 256-259** in the updated manuscript.

Comment 6: Results (Lines 270–380)
• Docking scores and interaction analyses are clearly presented.
• Line 320–340: Figures would benefit from clearer labels and more descriptive legends.

Our Response: We thank the reviewer for this helpful comment. We have revised the corresponding figures to improve label clarity and readability, including clearer residue names, bond types, and ligand identifiers. In addition, the figure legends have been expanded to provide more detailed descriptions of the docking poses and key protein–ligand interactions, allowing the figures to be interpreted independently of the main text. We have updated the labels and added more descriptive legends for figures cited in the mentioned lines. Please refer to legends of **Supplementary Fig. S1 and Supplementary Fig. S2** in the updated manuscript.

Comment 7: Discussion (Lines 390–470)
• The discussion appropriately contextualizes findings within existing literature.
• Concern: Some statements imply biological efficacy or clinical relevance that cannot be concluded from in silico analysis alone. These should be reframed as hypotheses for future experimental validation.

Our Response: We appreciate the reviewer's insightful comment. The Discussion section has been carefully revised to avoid implying biological efficacy or clinical relevance based solely on in silico analyses. Statements suggesting therapeutic or clinical potential have been rephrased to emphasize that our findings represent computational predictions and should be regarded as hypotheses requiring future in vitro and in vivo experimental validation. Please refer to the updated manuscript.

Comment 8: Conclusion (Lines 480–520)
• The conclusion summarizes the work effectively.
• Recommendation: Emphasize that the findings identify apigenin as a promising candidate for further experimental investigation, rather than as a confirmed inhibitor.

Our Response: We thank the reviewer for this valuable recommendation. The Conclusion section has been revised to emphasize that apigenin is identified as a promising candidate based on computational analyses and should be considered for further in vitro and in vivo experimental investigation, rather than being presented as a confirmed inhibitor. All statements implying definitive inhibitory or therapeutic effects have been rephrased accordingly.

Comment 9: Figures and Tables
• Figures are relevant but require improved resolution and annotation.
• All abbreviations should be defined in figure legends.

Our Response: We thank the reviewer for this helpful comment. All figures have been revised to improve resolution and visual clarity. In addition, figure annotations have been enhanced to ensure better readability and interpretation. All abbreviations used in the figures, tables and supplementary information are now explicitly defined in the corresponding legends.
