# Supplementary material for: In silico screening and molecular analyses identify apigenin from Scutellaria barbata as a potent AKT1 inhibitor in breast cancer
Source: PLoS One. 2026 Jun 25;21(6):e0338874. doi: 10.1371/journal.pone.0338874 (PMC13298910; doi:10.1371/journal.pone.0338874)
Supplement: Attachment — Submitted filename: Review Response.pdf [file pone.0338874.s020.pdf]

Journal Requirements:

1. If the reviewer comments include a recommendation to cite specific previously published works, please review and evaluate these publications to determine whether they are relevant and should be cited. There is no requirement to cite these works unless the editor has indicated otherwise.

Our response: We thank the editor for the suggestion. The recommended articles were carefully examined, and those directly relevant to our study have been cited in the revised manuscript.

2. Please review your reference list to ensure that it is complete and correct. If you have cited papers that have been retracted, please include the rationale for doing so in the manuscript text, or remove these references and replace them with relevant current references. Any changes to the reference list should be mentioned in the rebuttal letter that accompanies your revised manuscript. If you need to cite a retracted article, indicate the article's retracted status in the References list and also include a citation and full reference for the retraction notice.

Our response: Thank you for this concern. We have thoroughly checked the reference list to ensure that it is complete, accurate, and up to date. All references were verified for retraction status, and no retracted articles were cited in the manuscript.

6. Review Comments to the Author

Reviewer #2: I have accepted this manuscript in my previous revision, there is no more comments from my side..............................

Our response: We sincerely thank the reviewer for their positive evaluation of our manuscript and for confirming their acceptance in the previous revision. We greatly appreciate the reviewer's time and effort in reviewing our work and providing constructive feedback, which significantly contributed to improving the quality of the manuscript.

Reviewer #4: The work offers useful computational indications, but it remains preliminary since the conclusions are based on in-silico methods only. Experimental studies are still required to verify whether these predicted effects occur in real biological systems.

Our response: We agree with the reviewer # 4 that the present work provides useful computational indications but remains preliminary, as the conclusions are based exclusively on in silico analyses. Accordingly, experimental validation is required to determine whether the predicted effects are reproducible in biological systems. This limitation has now been explicitly stated in the manuscript, and experimental validation will be addressed in our future studies. Please refer to **Lines 677-688** in the updated manuscript.

Reviewer #5: It was a revised version of the manuscript. It seems the previous queries are supported and the work waits for finalization.

Our response: We thank Reviewer #5 for the encouraging comments on the revised manuscript. We are pleased to note that the previous concerns have been satisfactorily addressed. The manuscript has been carefully revised accordingly, and we believe it is now suitable for final consideration**.**
