# Supplementary material for: In silico screening and molecular analyses identify apigenin from Scutellaria barbata as a potent AKT1 inhibitor in breast cancer
Source: PLoS One. 2026 Jun 25;21(6):e0338874. doi: 10.1371/journal.pone.0338874 (PMC13298910; doi:10.1371/journal.pone.0338874)
Supplement: Attachment — Submitted filename: Response to Reviewers.pdf [file pone.0338874.s021.pdf]

Editorial/Journal Requirements

When submitting your revision, we need you to address these additional requirements.

Comment 1. Please ensure that your manuscript meets PLOS ONE meets style requirements, including those for file naming.

Our Response: Thank you for the comment. We have carefully reviewed the manuscript and revised it to ensure full compliance with PLOS ONE style requirements, including proper formatting and standardized file naming conventions for all submitted files.

Comment 2. Please provide additional details regarding participant consent. In the ethics statement in the Methods and online submission information, please ensure that you have specified (1) whether consent was informed and (2) what type you obtained (for instance, written or verbal, and if verbal, how it was documented and witnessed). If your study included minors, state whether you obtained consent from parents or guardians. If the need for consent was waived by the ethics committee, please include this information. If you are reporting a retrospective study of medical records or archived samples, please ensure that you have discussed whether all data were fully anonymized before you accessed them and/or whether the IRB or ethics committee waived the requirement for informed consent. If patients provided informed written consent to have data from their medical records used in research, please include this information.

Our Response: Thank you for your clarification. We have updated the ethics statement in the Methods section and online submission information to explicitly state that this study is entirely in silico and does not involve human participants, minors, patient data, medical records, or biological/archived samples. Therefore, no informed consent (written or verbal) was obtained or required, no consent documentation process was applicable, and no IRB/ethics committee approval or waiver of consent was necessary.

Comment 3. Thank you for stating the following financial disclosure:

"Ms J. Gavin awarded the FirstRand Bursary Trust. Ms J. Gavin awarded the National Research Foundation". Please state what role the funders took in the study. If the funders had no role, please state: "The funders had no role in study design, data collection and analysis, decision to publish, or preparation of the manuscript. If this statement is not correct you must amend it as needed. Please include this amended Role of Funder statement in your cover letter; we will change the online submission form on your behalf.

Our Response: Thank you for your comment. This study was conducted as an in-silico analysis and did not receive any direct project-specific funding from external donor agencies.

Comment 4. Thank you for stating the following in your Competing Interests section:

"None"

Please complete your Competing Interests on the online submission form to state any Competing Interests. If you have no competing interests, please state: The authors have declared that no competing interests exist. This information should be included in your cover letter; we will change the online submission form on your behalf.

Our Response: Thank you for your clarification. We have updated the Competing Interests statement in the manuscript and cover letter to read: *"The authors have declared that no competing interests exist."*

Comment 5. Please include your full ethics statement in the 'Methods' section of your manuscript file. In your statement, please include the full name of the IRB or ethics committee who approved or waived your study, as well as whether or not you obtained informed written or verbal consent. If consent was waived for your study, please include this information in your statement as well.

Our Response: Thank you for your clarification. We have added an ethics statement in the Methods section stating that this study is entirely in silico, involves no human or animal subjects, and therefore did not require approval from an Institutional Review Board or ethics committee, nor informed consent.

Comment 6. Please ensure that you refer to **Figure 5 to 8** in your text as, if accepted, production will need this reference to link the reader to the figure.

Our Response: Thank you for highlighting this. We have carefully reviewed the manuscript and ensured that all figures (**Figures 5–8**) are appropriately cited in the text to facilitate proper linkage during production.

Comment 7. Please include a separate caption for each figure in your manuscript.

Our Response: Thank you for the suggestion. We have revised the manuscript to include a separate caption for each figure, ensuring clarity and consistency throughout.

Comment 8. Please upload a copy of Figure 2, to which you refer in your text on page 10. If the figure is no longer to be included as part of the submission please remove all reference to it within the text.

Our Response: Thank you for pointing this out. We have now uploaded **Figure 2** as part of the revised submission and ensured that all in-text references to the figure are accurate and consistent.

Comment 9. Please include captions for your Supporting Information files at the end of your manuscript, and update any in-text citations to match accordingly.

Our Response: Thank you for the suggestion. We have added appropriate captions for all Supporting Information files at the end of the manuscript and revised the in-text citations to ensure consistency with these updates.

Comment 10. We notice that your supplementary tables are included in the manuscript file. Please remove them and upload them with the file type; Supporting Information; Please ensure that each Supporting Information file has a legend listed in the manuscript after the references list.

Our Response: Thank you for the guidance regarding the supplementary materials. We have removed the supplementary tables from the main manuscript and uploaded them separately as Supporting Information files. In addition, we have included appropriate legends for each file in the manuscript after the references section, as instructed.

Comment 11. If the reviewer comments include a recommendation to cite specific previously published works, please review and evaluate these publications to determine whether they are

relevant and should be cited. There is no requirement to cite these works unless the editor has indicated otherwise.

Our Response: We thank the reviewer for the suggestion to consider additional citations. We carefully evaluated the recommended publications for their relevance to our study and have incorporated those that are directly pertinent to our work. References that were not closely aligned with the scope or focus of this manuscript were not included.

Response to Reviewer #1 Comments

Overall Comment: This manuscript presents a timely and relevant synthesis of the complex roles of steroid hormones and their receptors in cancer biology. The topic is important and aligns well with current directions in precision oncology, particularly in understanding hormone-driven malignancies. The effort to integrate molecular mechanisms with epidemiological and clinical insights is commendable. However, several key areas require further clarification and strengthening before the manuscript can be considered for publication.

Our Response: We thank the reviewer for the constructive feedback and for recognizing the relevance of our work. In response, we have revised the manuscript to improve clarity, strengthen the discussion of molecular mechanisms, enhance integration with clinical evidence, and include updated references. We believe these changes have improved the overall quality of the manuscript.

Comment 1: Clarity of Study Design (Narrative vs Systematic Review)
#The manuscript alternates between describing itself as a "mini-review" and implying elements of a systematic review.
#Please clearly define the review type (e.g., narrative review, scoping review, or systematic review).
#If systematic elements are retained, include:

Search strategy (databases, keywords, time frame)
Inclusion/exclusion criteria
Study selection process (e.g., PRISMA flow diagram)

Our Response: We thank the reviewer for this important observation. We would like to clarify that the manuscript is original *in silico* computational study rather than a narrative or systematic review. The computational nature of the study has been clearly defined in the Title and Abstract, and the full methodological workflow is described in the Materials and Methods section (**page 1–15**).
To address the reviewer's concern regarding clarity, we have ensured that the study design is consistently described throughout the manuscript. The following information is already included in the revised version:

- Network pharmacology analysis
- Molecular docking analysis
- Pharmacophore modeling
- Molecular dynamics simulations
- Electronic structure and reactivity analysis

Accordingly, elements such as PRISMA flow diagrams, literature search strategies, and inclusion/exclusion criteria are not applicable to this study

Comment 2: Methodological Transparency
#The literature search methodology lacks sufficient detail.
#A dedicated Methods section should be included even for a narrative review to enhance reproducibility and transparency.

Our Response: We thank the reviewer for this valuable comment. We would like to clarify that the computational methodology, including network pharmacology, molecular docking, Pharmacophore modeling, molecular dynamics simulation, electronic structure and reactivity analysis are already fully described in the Materials and Methods section (**page 6–15**). We have further revised the section to improve clarity, structure, and reproducibility.

Comment 3: Critical Appraisal of Literature
#The manuscript currently summarizes findings effectively but lacks critical evaluation.
#Strengthen the discussion by:

Highlighting conflicting findings across studies
Discussing study limitations (e.g., sample size, bias, population differences)
Identifying gaps in current knowledge

Our Response: We appreciate this insightful suggestion. The Discussion section of the revised manuscript already includes a critical evaluation of the relevant literature. Specifically, it contains:
• Comparison with previously reported computational studies
• Discussion of consistent and conflicting findings across studies
• Identification of current knowledge gaps related to AKT1 inhibition and phytocompounds
These elements have been clearly incorporated in the revised Discussion section (**page 26–31**).

Comment 4: Data Availability Statement
#Although this is a review, PLOS ONE requires clarity regarding data sources.
#Include a statement specifying that all data are derived from published literature and provide supplementary material if applicable.

Our Response: We thank the reviewer for highlighting this requirement. The manuscript already includes a Data Availability Statement indicating that all data and computational parameters are included within the manuscript and Supporting Information files.

Comment 5: Limitations Section
#A dedicated section outlining the limitations of the review itself is needed.

This should include:
Potential publication bias
Database selection limitations
Language restrictions (if any)

Our Response: We agree with the reviewer that a clear limitations section is important. A dedicated "Limitations" section has included in the discussion section (**line no. 687-698**). This section addresses: the reliance on computational predictions and the absence of experimental (in vitro and in vivo) validation.

Minor Comments

Comment 1: Consistency in Terminology

#Ensure consistent use of terms such as "steroid hormones," "sex hormones," and "hormone-related receptors.

Our Response: The manuscript has been carefully reviewed and revised to ensure consistent terminology throughout.

Comment 2: Language and Readability
#Some sentences are overly complex and may reduce readability.
#Consider simplifying long sentences, especially in the Introduction and Discussion.

Our Response: The manuscript has been professionally edited to improve clarity, readability, and sentence structure.

Comment 3: Abbreviations
#Ensure all abbreviations are defined at first mention (e.g., ER, PR, AR).

Our Response: All abbreviations are already defined at first mention in the manuscript.

Comment 4: Figures and Tables

#Table 1 improvement is noted; however:

Ensure all entries are referenced

Consider adding a brief explanatory legend

#Figures illustrating hormone-receptor signaling pathways would enhance clarity

Our Response: We thank the reviewer for highlighting this requirement. The manuscript has been carefully reviewed and revised to ensure consistent legends of Figures and Tables.

Response to Reviewer #6 Comments

Comment 1: The manuscript identifies several phytocompounds from *S. barbata* targeting breast cancer (BC) oncogenesis pathways using computational approaches. In particular, apigenin is highlighted as a potential candidate for modulating cancer signaling pathways. However, the conclusions are mainly based on in silico analyses, and further experimental validation is needed to confirm these findings.

The language in this manuscript is generally correct, but it is sometimes difficult to follow. The manuscript is also overly long and lacks a clear focus on the main points. In addition, some figures are of poor resolution (e.g., Fig. 1) and should be improved.

Our Response: We thank the reviewer for the constructive comments. We have revised the manuscript to improve clarity, readability, structure, and figure quality. We acknowledge that the findings are based on *in-silico* analyses (network pharmacology, molecular docking, and molecular dynamics simulations), and further experimental validation is largely necessary, and we have acknowledged this limitation in abstract (**page line no. 45-46)** and discussion section (**page line no. 687-698**). The manuscript has also been

edited to improve language clarity, reduce redundancy, and enhance flow, with the discussion streamlined to focus on key findings, particularly the AKT1–apigenin interaction. We have carefully reviewed all figures and **Figure 1**, have been replaced with high-resolution versions (600 dpi) to improve quality and readability.
