# Supplementary material for: In silico screening and molecular analyses identify apigenin from Scutellaria barbata as a potent AKT1 inhibitor in breast cancer
Source: PLoS One. 2026 Jun 25;21(6):e0338874. doi: 10.1371/journal.pone.0338874 (PMC13298910; doi:10.1371/journal.pone.0338874)
Supplement: Peer review history

# Peer review: Decision Letter 0

**DOI:** 10.1371/journal.pone.0338874.r001

**Authors:** Zhiling Yu

26 Dec 2025

PONE-D-25-63895

Computational Screening Identifies Apigenin from Scutellaria barbata as a Potent AKT1 Inhibitor in Breast Cancer

PLOS One

Dear Dr. Hoque,

Thank you for submitting your manuscript to PLOS ONE. After careful consideration, we feel that it has merit but does not fully meet PLOS ONE’s publication criteria as it currently stands. Therefore, we invite you to submit a revised version of the manuscript that addresses the points raised during the review process.

Please submit your revised manuscript by Feb 09 2026 11:59PM. If you will need more time than this to complete your revisions, please reply to this message or contact the journal office at plosone@plos.org. When you’re ready to submit your revision, log on to https://www.editorialmanager.com/pone/ and select the 'Submissions Needing Revision' folder to locate your manuscript file.

Please include the following items when submitting your revised manuscript:

- A letter that responds to each point raised by the academic editor and reviewer(s). You should upload this letter as a separate file labeled 'Response to Reviewers'.
- A marked-up copy of your manuscript that highlights changes made to the original version. You should upload this as a separate file labeled 'Revised Manuscript with Track Changes'.
- An unmarked version of your revised paper without tracked changes. You should upload this as a separate file labeled 'Manuscript'.

If you would like to make changes to your financial disclosure, please include your updated statement in your cover letter. Guidelines for resubmitting your figure files are available below the reviewer comments at the end of this letter.

If applicable, we recommend that you deposit your laboratory protocols in protocols.io to enhance the reproducibility of your results. Protocols.io assigns your protocol its own identifier (DOI) so that it can be cited independently in the future. For instructions see: https://journals.plos.org/plosone/s/submission-guidelines#loc-laboratory-protocols. Additionally, PLOS ONE offers an option for publishing peer-reviewed Lab Protocol articles, which describe protocols hosted on protocols.io. Read more information on sharing protocols at https://plos.org/protocols?utm_medium=editorial-email&utm_source=authorletters&utm_campaign=protocols.

We look forward to receiving your revised manuscript.

Kind regards,

Zhiling Yu

Academic Editor

PLOS One

Journal Requirements:

When submitting your revision, we need you to address these additional requirements.

1. Please ensure that your manuscript meets PLOS ONE's style requirements, including those for file naming. The PLOS ONE style templates can be found at

https://journals.plos.org/plosone/s/file?id=wjVg/PLOSOne_formatting_sample_main_body.pdf and

https://journals.plos.org/plosone/s/file?id=ba62/PLOSOne_formatting_sample_title_authors_affiliations.pdf

2. Please note that PLOS One has specific guidelines on code sharing for submissions in which author-generated code underpins the findings in the manuscript. In these cases, we expect all author-generated code to be made available without restrictions upon publication of the work. Please review our guidelines at https://journals.plos.org/plosone/s/materials-and-software-sharing#loc-sharing-code and ensure that your code is shared in a way that follows best practice and facilitates reproducibility and reuse.

3. Please upload a copy of Figure 8, to which you refer in your text on page 23. If the figure is no longer to be included as part of the submission please remove all reference to it within the text.

4. If the reviewer comments include a recommendation to cite specific previously published works, please review and evaluate these publications to determine whether they are relevant and should be cited. There is no requirement to cite these works unless the editor has indicated otherwise.

[Note: HTML markup is below. Please do not edit.]

Reviewers' comments:

Reviewer's Responses to Questions

**Comments to the Author**

1. Is the manuscript technically sound, and do the data support the conclusions?

The manuscript must describe a technically sound piece of scientific research with data that supports the conclusions. Experiments must have been conducted rigorously, with appropriate controls, replication, and sample sizes. The conclusions must be drawn appropriately based on the data presented.

Reviewer #1: Partly

Reviewer #2: Yes

Reviewer #3: Partly

**********

2. Has the statistical analysis been performed appropriately and rigorously?

Reviewer #1: N/A

Reviewer #2: N/A

Reviewer #3: Yes

**********

3. Have the authors made all data underlying the findings in their manuscript fully available?

The PLOS Data policy requires authors to make all data underlying the findings described in their manuscript fully available without restriction, with rare exception (please refer to the Data Availability Statement in the manuscript PDF file). The data should be provided as part of the manuscript or its supporting information, or deposited to a public repository. For example, in addition to summary statistics, the data points behind means, medians and variance measures should be available. If there are restrictions on publicly sharing data—e.g. participant privacy or use of data from a third party—those must be specified.

Reviewer #1: Yes

Reviewer #2: Yes

Reviewer #3: Yes

**********

4. Is the manuscript presented in an intelligible fashion and written in standard English?

PLOS ONE does not copyedit accepted manuscripts, so the language in submitted articles must be clear, correct, and unambiguous. Any typographical or grammatical errors should be corrected at revision, so please note any specific errors here.

Reviewer #1: Yes

Reviewer #2: Yes

Reviewer #3: Yes

**********

5. Review Comments to the Author

Please use the space provided to explain your answers to the questions above. You may also include additional comments for the author, including concerns about dual publication, research ethics, or publication ethics. (Please upload your review as an attachment if it exceeds 20,000 characters)

Reviewer #1: Thank you for the opportunity to review this interesting manuscript exploring the anticancer potential of Scutellaria barbata through a comprehensive in silico workflow. The integration of network pharmacology, molecular docking, MD simulations, MM-GBSA, and DFT analyses presents a strong computational framework. The topic is timely, and the manuscript has the potential to contribute meaningfully to natural-product–based drug discovery in breast cancer research.

However, several substantive issues should be addressed before the manuscript can be considered for publication. My detailed comments follow.

Major Comments

1. Overinterpretation of computational findings (Lines ~38–41, 546–612)

The manuscript frequently implies definitive biological activity based solely on computational evidence. PLOS ONE requires that conclusions be supported strictly by the presented data. Statements such as “apigenin is a promising multi-target flavonoid for combating breast cancer” (Line 38) or references to “therapeutic potential” should be tempered.

Recommendation: Reframe conclusions to emphasize predictive or hypothesis-generating nature of the study.

2. Lack of clarity in methodology and insufficient reproducibility in several sections

Although the Methods section is detailed overall, certain areas require clarification to ensure reproducibility:

a. Dataset reproducibility (Lines 289–307, 331–340)

• The authors list total numbers of genes/targets, but the raw gene lists are not provided in supplementary files.

• It is unclear how duplicate genes were removed (based on gene symbol, UniProt ID, Entrez ID?).

b. Docking methodology (Lines 213–227)

• Grid box dimensions are listed, but grid box centers (x,y,z) are not fully specified for all proteins.

• Justification for control drugs (resveratrol, tartaric acid, etc.) should be strengthened, as some are not standard inhibitors of the target proteins.

c. MD simulations (Lines 241–258)

• The RMSD and RMSF analyses are descriptive, but statistical summaries (mean ± SD for stable intervals) would help interpretation.

• Figures should show ligand-only RMSD, not only protein–ligand complex RMSD.

3. Network pharmacology interpretation requires refinement (Lines 344–384)

The enrichment results include many infection-related pathways (e.g., EBV, hepatitis B/C). These are common artifacts of over-broad gene lists.

Recommendation:

• Authors should justify inclusion of these pathways or filter KEGG pathways more stringently.

• Alternatively, reorganize KEGG results to focus on cancer-specific and signal-transduction–related pathways.

4. Hub protein selection requires more rigorous justification (Lines 386–401)

While the use of five CytoHubba algorithms is commendable, the integration strategy is unclear.

Specific gaps:

• The manuscript does not explain how the four proteins were chosen when multiple algorithms produced larger sets.

• No statistical robustness measure is provided.

Recommendation:

Include a transparent explanation of the decision rule (e.g., “proteins appearing in ≥3 top-10 lists were considered hubs”).

5. English expression, clarity, and grammar need improvement (Lines 48–120, 546–612)

The manuscript reads clearly in many sections, but substantial grammatical and stylistic issues remain:

Examples:

• Line 67: “Conventional BC therapies continue to face major limitations that compromise treatment efficacy and patient well-being.” (Fragmentary transition)

• Line 120: “This multilayered computational approach bridges traditional empirical knowledge…” (awkward phrasing)

Recommendation:

Professional language revision is advised to improve readability.

Minor Comments

1. Figures

o Some figure legends are incomplete (particularly Figs. 6 and 7).

o Complex figures (e.g., S3–S4) require higher resolution.

2. Units and formatting

o In some cases, spacing between numbers and units is inconsistent (e.g., “100 ns”, “0.14838 a.u.”).

o Please ensure uniform use of italic formatting for variables such as p, η, S, etc.

3. Toxicity classification (Lines 289–307)

o The authors mention “class 4 and 5” toxicity but do not provide the criteria for these classes.

o Please reference OECD or ProTox categories explicitly.

4. References

o Some citations are outdated given the rapidly evolving field of computational oncology.

o Please update key background references (e.g., network pharmacology, MD in drug discovery).

Reviewer #2: The article is presented well, it clearly show the computational techniques used for the identification of apigenin from target plant against cancer, the manuscript is well presented and can be accepted, however following points needs to be addressed well.

1. What does it meant by “compounds lacking SMILES identifiers” ? if the compounds have no smiles ,so they were removed? Please explain.

2. Line 163 and onward, it seems confusion here, the authors try PPI, which is normally among the overlapping genes between the disease and compounds, then the authors add here compounds, please recheck this description.

3. The binding scores are reported in the discussion, but it doesn't critically compare these compounds with established breast cancer drugs or known P13K/AKT inhibitors, further references are needed to support the claim.

4. The GO and KEGG pathway enrichment results largely repeate well-known cancer pathways, displaying limited novel mechanistic understanding specific to breast cancer subtypes.

5. The introduction should be further strengthened by the addition of importance of network pharmacology and molecular docking, the author can take information form these references or any other relevant one (PMID: 40764661, PMID: 41096765)

6. Please provide all the relevant supporting information regarding network pharmacology, it should be the data obtained from different databases.

Reviewer #3: Major Comments

1. Network pharmacology and KEGG analyses identify largely generic cancer and inflammatory pathways. Greater emphasis on breast cancer–specific relevance and mechanistic prioritization is needed.

2. Molecular dynamics simulations were carried out only for AKT1, even though several key target proteins were identified. Authors are requested to perform dynamics for other targets proteins as well.

3. Target prioritization is insufficiently justified. Although multiple hub proteins were identified, the rationale for emphasizing AKT1—and apigenin in particular—is unclear, especially since hispidulin shows stronger binding in docking and MM-GBSA analyses. Thus, authors are requested to give information on this point as well.

4. Compound filtering based on ADME thresholds (OB, DL) may bias selection and exclude potentially active phytochemicals. The authors are requested to give clear justification for their choice of cut-offs.

5. Entropy was not included in the MM-GBSA calculations; the authors should explain this omission and discuss how it may affect the interpretation of the binding energy results.

Minor Comments

1. Abbreviations (e.g., RA, AEs, SAEs, DMARDs) should be defined at first mention in the main text and used consistently thereafter.

2. Minor inconsistencies in referencing style and citation formatting should be corrected.

3. The resolution and figure legend of several figures should be improved for better understanding and readability.

4. Minor inconsistencies in referencing style and citation formatting should be corrected to align with the journal’s guidelines.

**********

6. PLOS authors have the option to publish the peer review history of their article (what does this mean?). If published, this will include your full peer review and any attached files.

If you choose “no”, your identity will remain anonymous but your review may still be made public.

**Do you want your identity to be public for this peer review?**  For information about this choice, including consent withdrawal, please see our Privacy Policy.

Reviewer #1: **Yes:**  Saheed Ayodeji Adekola

Reviewer #2: No

Reviewer #3: **Yes:**  Mirav Patel

**********

[NOTE: If reviewer comments were submitted as an attachment file, they will be attached to this email and accessible via the submission site. Please log into your account, locate the manuscript record, and check for the action link "View Attachments". If this link does not appear, there are no attachment files.]

To ensure your figures meet our technical requirements, please review our figure guidelines: https://journals.plos.org/plosone/s/figures

You may also use PLOS’s free figure tool, NAAS, to help you prepare publication quality figures: https://journals.plos.org/plosone/s/figures#loc-tools-for-figure-preparation.

NAAS will assess whether your figures meet our technical requirements by comparing each figure against our figure specifications.

# Author comment: Author response to Decision Letter 1

**DOI:** 10.1371/journal.pone.0338874.r002

15 Jan 2026

We have revised and updated the manuscript according to reviewers/editor's suggestions. Please refer to the revised submission.

Attachment Submitted filename: Responses to Reviewers.pdf

# Peer review: Decision Letter 1

**DOI:** 10.1371/journal.pone.0338874.r003

**Authors:** Zhiling Yu, Zhiling Yu

30 Jan 2026

PONE-D-25-63895R1

Computational Screening Identifies Apigenin from Scutellaria barbata as a Potent AKT1 Inhibitor in Breast Cancer

PLOS One

Dear Dr. Hoque,

Thank you for submitting your manuscript to PLOS ONE. After careful consideration, we feel that it has merit but does not fully meet PLOS ONE’s publication criteria as it currently stands. Therefore, we invite you to submit a revised version of the manuscript that addresses the points raised during the review process.

Please submit your revised manuscript by Mar 16 2026 11:59PM. If you will need more time than this to complete your revisions, please reply to this message or contact the journal office at plosone@plos.org. When you're ready to submit your revision, log on to https://www.editorialmanager.com/pone/ and select the 'Submissions Needing Revision' folder to locate your manuscript file.

Please include the following items when submitting your revised manuscript:

- A letter that responds to each point raised by the academic editor and reviewer(s). You should upload this letter as a separate file labeled 'Response to Reviewers'.
- A marked-up copy of your manuscript that highlights changes made to the original version. You should upload this as a separate file labeled 'Revised Manuscript with Track Changes'.
- An unmarked version of your revised paper without tracked changes. You should upload this as a separate file labeled 'Manuscript'.

If you would like to make changes to your financial disclosure, please include your updated statement in your cover letter. Guidelines for resubmitting your figure files are available below the reviewer comments at the end of this letter.

If applicable, we recommend that you deposit your laboratory protocols in protocols.io to enhance the reproducibility of your results. Protocols.io assigns your protocol its own identifier (DOI) so that it can be cited independently in the future. For instructions see: https://journals.plos.org/plosone/s/submission-guidelines#loc-laboratory-protocols. Additionally, PLOS ONE offers an option for publishing peer-reviewed Lab Protocol articles, which describe protocols hosted on protocols.io. Read more information on sharing protocols at https://plos.org/protocols?utm_medium=editorial-email&utm_source=authorletters&utm_campaign=protocols.

We look forward to receiving your revised manuscript.

Kind regards,

Editor

PLOS One

**Journal Requirements:**

1. If the reviewer comments include a recommendation to cite specific previously published works, please review and evaluate these publications to determine whether they are relevant and should be cited. There is no requirement to cite these works unless the editor has indicated otherwise.

[Note: HTML markup is below. Please do not edit.]

Reviewers' comments:

Reviewer's Responses to Questions

**Comments to the Author**

1. If the authors have adequately addressed your comments raised in a previous round of review and you feel that this manuscript is now acceptable for publication, you may indicate that here to bypass the “Comments to the Author” section, enter your conflict of interest statement in the “Confidential to Editor” section, and submit your "Accept" recommendation.

Reviewer #1: All comments have been addressed

Reviewer #2: All comments have been addressed

**********

2. Is the manuscript technically sound, and do the data support the conclusions?

The manuscript must describe a technically sound piece of scientific research with data that supports the conclusions. Experiments must have been conducted rigorously, with appropriate controls, replication, and sample sizes. The conclusions must be drawn appropriately based on the data presented.

Reviewer #1: Partly

Reviewer #2: Yes

**********

3. Has the statistical analysis been performed appropriately and rigorously?

Reviewer #1: N/A

Reviewer #2: N/A

**********

4. Have the authors made all data underlying the findings in their manuscript fully available?

The PLOS Data policy requires authors to make all data underlying the findings described in their manuscript fully available without restriction, with rare exception (please refer to the Data Availability Statement in the manuscript PDF file). The data should be provided as part of the manuscript or its supporting information, or deposited to a public repository. For example, in addition to summary statistics, the data points behind means, medians and variance measures should be available. If there are restrictions on publicly sharing data—e.g. participant privacy or use of data from a third party—those must be specified.

Reviewer #1: Yes

Reviewer #2: Yes

**********

5. Is the manuscript presented in an intelligible fashion and written in standard English?

PLOS ONE does not copyedit accepted manuscripts, so the language in submitted articles must be clear, correct, and unambiguous. Any typographical or grammatical errors should be corrected at revision, so please note any specific errors here.

Reviewer #1: Yes

Reviewer #2: Yes

**********

6. Review Comments to the Author

Please use the space provided to explain your answers to the questions above. You may also include additional comments for the author, including concerns about dual publication, research ethics, or publication ethics. (Please upload your review as an attachment if it exceeds 20,000 characters)

Reviewer #1: This manuscript presents a computational investigation into apigenin as a putative AKT1 inhibitor, contributing to the growing literature on phytochemicals as potential anticancer agents. The study is relevant, clearly motivated, and methodologically coherent; however, several areas require clarification and strengthening before the conclusions can be fully supported.

Title (Lines 1–2)

• The title is clear and accurately reflects the computational nature of the study.

• Suggestion: Consider adding “in silico” to the title to avoid overinterpretation of biological efficacy.

Abstract (Lines 20–45)

• The abstract summarizes the study well.

• Concern: Phrases suggesting therapeutic potency should be tempered to reflect computational prediction rather than experimental validation.

Introduction (Lines 50–130)

• The background on AKT1 signaling and breast cancer is well articulated.

• Line 95–110: The rationale for selecting Scutellaria barbata and apigenin should be more explicitly linked to prior experimental or clinical evidence.

Materials and Methods (Lines 140–260)

• Docking and computational tools are appropriately selected.

• Line 175–190: Protein and ligand preparation steps require more detail to ensure reproducibility.

• Please specify docking validation procedures (e.g., re-docking, RMSD thresholds).

Results (Lines 270–380)

• Docking scores and interaction analyses are clearly presented.

• Line 320–340: Figures would benefit from clearer labels and more descriptive legends.

Discussion (Lines 390–470)

• The discussion appropriately contextualizes findings within existing literature.

• Concern: Some statements imply biological efficacy or clinical relevance that cannot be concluded from in silico analysis alone. These should be reframed as hypotheses for future experimental validation.

Conclusion (Lines 480–520)

• The conclusion summarizes the work effectively.

• Recommendation: Emphasize that the findings identify apigenin as a promising candidate for further experimental investigation, rather than as a confirmed inhibitor.

Figures and Tables

• Figures are relevant but require improved resolution and annotation.

• All abbreviations should be defined in figure legends.

Ethics and Publication Concerns

• No ethical or publication integrity concerns were identified.

Reviewer #2: The authors have revised the manuscript well........................................................

**********

7. PLOS authors have the option to publish the peer review history of their article (what does this mean?). If published, this will include your full peer review and any attached files.

If you choose “no”, your identity will remain anonymous but your review may still be made public.

**Do you want your identity to be public for this peer review?**  For information about this choice, including consent withdrawal, please see our Privacy Policy.

Reviewer #1: **Yes:**  Saheed Ayodeji Adekola

Reviewer #2: No

**********

[NOTE: If reviewer comments were submitted as an attachment file, they will be attached to this email and accessible via the submission site. Please log into your account, locate the manuscript record, and check for the action link "View Attachments". If this link does not appear, there are no attachment files.]

To ensure your figures meet our technical requirements, please review our figure guidelines: https://journals.plos.org/plosone/s/figures

You may also use PLOS’s free figure tool, NAAS, to help you prepare publication quality figures: https://journals.plos.org/plosone/s/figures#loc-tools-for-figure-preparation.

NAAS will assess whether your figures meet our technical requirements by comparing each figure against our figure specifications.

# Author comment: Author response to Decision Letter 2

**DOI:** 10.1371/journal.pone.0338874.r004

1 Feb 2026

Dear Editor,

Thank you for the editor's decision letter dated on January 31, 2026. Enclosed within this submission is our point-by-point responses to the comments raised by both reviewers/editors. We would like to take this opportunity to express our sincere thanks to the expert reviewers/editors who identified several areas in our manuscript that were needed corrections as well as modifications. We also would like to cordially thank you for allowing us the change to resubmit a revised version of the manuscript.

We have revised the title of the manuscript according reviewer’s suggestion, and updated the manuscript based on suggestions from the reviewers, incorporating several modifications. Please find all changes highlighted in RED color fonts in the revised manuscript. We also have provided a clean manuscript for your kind perusal.

Attachment Submitted filename: Responses_to_Reviewers_auresp_2.pdf

# Peer review: Decision Letter 2

**DOI:** 10.1371/journal.pone.0338874.r005

**Authors:** Zhiling Yu, Zhiling Yu, Zhiling Yu

3 Mar 2026

PONE-D-25-63895R2

In Silico Screening and Molecular Analyses Identify Apigenin from Scutellaria barbata as a Potent AKT1 Inhibitor in Breast Cancer

PLOS One

Dear Dr. Hoque,

Thank you for submitting your manuscript to PLOS ONE. After careful consideration, we feel that it has merit but does not fully meet PLOS ONE’s publication criteria as it currently stands. Therefore, we invite you to submit a revised version of the manuscript that addresses the points raised during the review process.

Please submit your revised manuscript by Apr 17 2026 11:59PM. If you will need more time than this to complete your revisions, please reply to this message or contact the journal office at plosone@plos.org. When you're ready to submit your revision, log on to https://www.editorialmanager.com/pone/ and select the 'Submissions Needing Revision' folder to locate your manuscript file.

Please include the following items when submitting your revised manuscript:

- A letter that responds to each point raised by the academic editor and reviewer(s). You should upload this letter as a separate file labeled 'Response to Reviewers'.
- A marked-up copy of your manuscript that highlights changes made to the original version. You should upload this as a separate file labeled 'Revised Manuscript with Track Changes'.
- An unmarked version of your revised paper without tracked changes. You should upload this as a separate file labeled 'Manuscript'.

-->If you would like to make changes to your financial disclosure, please include your updated statement in your cover letter. Guidelines for resubmitting your figure files are available below the reviewer comments at the end of this letter.

If applicable, we recommend that you deposit your laboratory protocols in protocols.io to enhance the reproducibility of your results. Protocols.io assigns your protocol its own identifier (DOI) so that it can be cited independently in the future. For instructions see: https://journals.plos.org/plosone/s/submission-guidelines#loc-laboratory-protocols. Additionally, PLOS ONE offers an option for publishing peer-reviewed Lab Protocol articles, which describe protocols hosted on protocols.io. Read more information on sharing protocols at https://plos.org/protocols?utm_medium=editorial-email&utm_source=authorletters&utm_campaign=protocols.

We look forward to receiving your revised manuscript.

Kind regards,

Editor

PLOS One

Journal Requirements:

1. If the reviewer comments include a recommendation to cite specific previously published works, please review and evaluate these publications to determine whether they are relevant and should be cited. There is no requirement to cite these works unless the editor has indicated otherwise.

2. Please review your reference list to ensure that it is complete and correct. If you have cited papers that have been retracted, please include the rationale for doing so in the manuscript text, or remove these references and replace them with relevant current references. Any changes to the reference list should be mentioned in the rebuttal letter that accompanies your revised manuscript. If you need to cite a retracted article, indicate the article’s retracted status in the References list and also include a citation and full reference for the retraction notice.

[Note: HTML markup is below. Please do not edit.]

Reviewers' comments:

Reviewer's Responses to Questions

**Comments to the Author**

1. If the authors have adequately addressed your comments raised in a previous round of review and you feel that this manuscript is now acceptable for publication, you may indicate that here to bypass the “Comments to the Author” section, enter your conflict of interest statement in the “Confidential to Editor” section, and submit your "Accept" recommendation.

Reviewer #2: All comments have been addressed

Reviewer #4: All comments have been addressed

Reviewer #5: All comments have been addressed

**********

2. Is the manuscript technically sound, and do the data support the conclusions?

The manuscript must describe a technically sound piece of scientific research with data that supports the conclusions. Experiments must have been conducted rigorously, with appropriate controls, replication, and sample sizes. The conclusions must be drawn appropriately based on the data presented.

Reviewer #2: Yes

Reviewer #4: Yes

Reviewer #5: Partly

**********

3. Has the statistical analysis been performed appropriately and rigorously?

Reviewer #2: Yes

Reviewer #4: Yes

Reviewer #5: Yes

**********

4. Have the authors made all data underlying the findings in their manuscript fully available?

The PLOS Data policy requires authors to make all data underlying the findings described in their manuscript fully available without restriction, with rare exception (please refer to the Data Availability Statement in the manuscript PDF file). The data should be provided as part of the manuscript or its supporting information, or deposited to a public repository. For example, in addition to summary statistics, the data points behind means, medians and variance measures should be available. If there are restrictions on publicly sharing data—e.g. participant privacy or use of data from a third party—those must be specified.

Reviewer #2: Yes

Reviewer #4: Yes

Reviewer #5: Yes

**********

5. Is the manuscript presented in an intelligible fashion and written in standard English?

PLOS ONE does not copyedit accepted manuscripts, so the language in submitted articles must be clear, correct, and unambiguous. Any typographical or grammatical errors should be corrected at revision, so please note any specific errors here.

Reviewer #2: Yes

Reviewer #4: Yes

Reviewer #5: Yes

**********

6. Review Comments to the Author

Please use the space provided to explain your answers to the questions above. You may also include additional comments for the author, including concerns about dual publication, research ethics, or publication ethics. (Please upload your review as an attachment if it exceeds 20,000 characters)

Reviewer #2: I have accepted this manuscript in my previous revision, there is no more comments from my side...............................

Reviewer #4: The work offers useful computational indications, but it remains preliminary since the conclusions are based on in-silico methods only. Experimental studies are still required to verify whether these predicted effects occur in real biological systems.

Reviewer #5: It was a revised version of the manuscript. It seems the previous queries are supported and the work waits for finalization.

**********

7. PLOS authors have the option to publish the peer review history of their article (what does this mean?). If published, this will include your full peer review and any attached files.

If you choose “no”, your identity will remain anonymous but your review may still be made public.

**Do you want your identity to be public for this peer review?**  For information about this choice, including consent withdrawal, please see our Privacy Policy.

Reviewer #2: No

Reviewer #4: No

Reviewer #5: No

**********

[NOTE: If reviewer comments were submitted as an attachment file, they will be attached to this email and accessible via the submission site. Please log into your account, locate the manuscript record, and check for the action link "View Attachments". If this link does not appear, there are no attachment files.]

To ensure your figures meet our technical requirements, please review our figure guidelines: https://journals.plos.org/plosone/s/figures

You may also use PLOS’s free figure tool, NAAS, to help you prepare publication quality figures: https://journals.plos.org/plosone/s/figures#loc-tools-for-figure-preparation.

NAAS will assess whether your figures meet our technical requirements by comparing each figure against our figure specifications.

# Author comment: Author response to Decision Letter 3

**DOI:** 10.1371/journal.pone.0338874.r006

6 Mar 2026

Journal Requirements:

1. If the reviewer comments include a recommendation to cite specific previously published works, please review and evaluate these publications to determine whether they are relevant and should be cited. There is no requirement to cite these works unless the editor has indicated otherwise.

Our response: We thank the editor for the suggestion. The recommended articles were carefully examined, and those directly relevant to our study have been cited in the revised manuscript.

2. Please review your reference list to ensure that it is complete and correct. If you have cited papers that have been retracted, please include the rationale for doing so in the manuscript text, or remove these references and replace them with relevant current references. Any changes to the reference list should be mentioned in the rebuttal letter that accompanies your revised manuscript. If you need to cite a retracted article, indicate the article’s retracted status in the References list and also include a citation and full reference for the retraction notice.

Our response: Thank you for this concern. We have thoroughly checked the reference list to ensure that it is complete, accurate, and up to date. All references were verified for retraction status, and no retracted articles were cited in the manuscript.

6. Review Comments to the Author

Reviewer #2: I have accepted this manuscript in my previous revision, there is no more comments from my side...............................

Our response: We sincerely thank the reviewer for their positive evaluation of our manuscript and for confirming their acceptance in the previous revision. We greatly appreciate the reviewer’s time and effort in reviewing our work and providing constructive feedback, which significantly contributed to improving the quality of the manuscript.

Reviewer #4: The work offers useful computational indications, but it remains preliminary since the conclusions are based on in-silico methods only. Experimental studies are still required to verify whether these predicted effects occur in real biological systems.

Our response: We agree with the reviewer # 4 that the present work provides useful computational indications but remains preliminary, as the conclusions are based exclusively on in silico analyses. Accordingly, experimental validation is required to determine whether the predicted effects are reproducible in biological systems. This limitation has now been explicitly stated in the manuscript, and experimental validation will be addressed in our future studies. Please refer to Lines 677-688 in the updated manuscript.

Reviewer #5: It was a revised version of the manuscript. It seems the previous queries are supported and the work waits for finalization.

Our response: We thank Reviewer #5 for the encouraging comments on the revised manuscript. We are pleased to note that the previous concerns have been satisfactorily addressed. The manuscript has been carefully revised accordingly, and we believe it is now suitable for final consideration.

Attachment Submitted filename: Review Response.pdf

# Peer review: Decision Letter 3

**DOI:** 10.1371/journal.pone.0338874.r007

**Authors:** Zhiling Yu, Zhiling Yu, Zhiling Yu, Zhiling Yu

8 Apr 2026

PONE-D-25-63895R3

In Silico Screening and Molecular Analyses Identify Apigenin from Scutellaria barbata as a Potent AKT1 Inhibitor in Breast Cancer

PLOS One

Dear Dr. Hoque,

Thank you for submitting your manuscript to PLOS ONE. After careful consideration, we feel that it has merit but does not fully meet PLOS ONE’s publication criteria as it currently stands. Therefore, we invite you to submit a revised version of the manuscript that addresses the points raised during the review process.

Please submit your revised manuscript by May 23 2026 11:59PM. If you will need more time than this to complete your revisions, please reply to this message or contact the journal office at plosone@plos.org. When you're ready to submit your revision, log on to https://www.editorialmanager.com/pone/ and select the 'Submissions Needing Revision' folder to locate your manuscript file.

Please include the following items when submitting your revised manuscript:

- A letter that responds to each point raised by the academic editor and reviewer(s). You should upload this letter as a separate file labeled 'Response to Reviewers'.
- A marked-up copy of your manuscript that highlights changes made to the original version. You should upload this as a separate file labeled 'Revised Manuscript with Track Changes'.
- An unmarked version of your revised paper without tracked changes. You should upload this as a separate file labeled 'Manuscript'.

If you would like to make changes to your financial disclosure, please include your updated statement in your cover letter. Guidelines for resubmitting your figure files are available below the reviewer comments at the end of this letter.

If applicable, we recommend that you deposit your laboratory protocols in protocols.io to enhance the reproducibility of your results. Protocols.io assigns your protocol its own identifier (DOI) so that it can be cited independently in the future. For instructions see: https://journals.plos.org/plosone/s/submission-guidelines#loc-laboratory-protocols. Additionally, PLOS ONE offers an option for publishing peer-reviewed Lab Protocol articles, which describe protocols hosted on protocols.io. Read more information on sharing protocols at https://plos.org/protocols?utm_medium=editorial-email&utm_source=authorletters&utm_campaign=protocols.

As the corresponding author, your ORCID iD is verified in the submission system and will appear in the published article. PLOS supports the use of ORCID, and we encourage all coauthors to register for an ORCID iD and use it as well. Please encourage your coauthors to verify their ORCID iD within the submission system before final acceptance, as unverified ORCID iDs will not appear in the published article. *Only*  the individual author can complete the verification step; PLOS staff *cannot*  verify ORCID iDs on behalf of authors.

We look forward to receiving your revised manuscript.

Kind regards,

Editor

PLOS One

Journal Requirements:

When submitting your revision, we need you to address these additional requirements.

1. Please ensure that your manuscript meets PLOS ONE's style requirements, including those for file naming. The PLOS ONE style templates can be found at

https://journals.plos.org/plosone/s/file?id=wjVg/PLOSOne_formatting_sample_main_body.pdf and

https://journals.plos.org/plosone/s/file?id=ba62/PLOSOne_formatting_sample_title_authors_affiliations.pdf

2. Please provide additional details regarding participant consent. In the ethics statement in the Methods and online submission information, please ensure that you have specified (1) whether consent was informed and (2) what type you obtained (for instance, written or verbal, and if verbal, how it was documented and witnessed). If your study included minors, state whether you obtained consent from parents or guardians. If the need for consent was waived by the ethics committee, please include this information.

If you are reporting a retrospective study of medical records or archived samples, please ensure that you have discussed whether all data were fully anonymized before you accessed them and/or whether the IRB or ethics committee waived the requirement for informed consent. If patients provided informed written consent to have data from their medical records used in research, please include this information.

3. Thank you for stating the following financial disclosure:

“Ms J. Gavin awarded the FirstRand Bursary Trust

Ms J. Gavin awarded the National Research Foundation”

Please state what role the funders took in the study. If the funders had no role, please state: "The funders had no role in study design, data collection and analysis, decision to publish, or preparation of the manuscript."

If this statement is not correct you must amend it as needed.

Please include this amended Role of Funder statement in your cover letter; we will change the online submission form on your behalf.

4. Thank you for stating the following in your Competing Interests section:

“None”

Please complete your Competing Interests on the online submission form to state any Competing Interests. If you have no competing interests, please state "The authors have declared that no competing interests exist.", as detailed online in our guide for authors at http://journals.plos.org/plosone/s/submit-now

This information should be included in your cover letter; we will change the online submission form on your behalf.

5. Please include your full ethics statement in the ‘Methods’ section of your manuscript file. In your statement, please include the full name of the IRB or ethics committee who approved or waived your study, as well as whether or not you obtained informed written or verbal consent. If consent was waived for your study, please include this information in your statement as well.

6. Please ensure that you refer to Figure 5 to 28 in your text as, if accepted, production will need this reference to link the reader to the figure.

7. Please include a separate caption for each figure in your manuscript.

8. Please upload a copy of Figure 2, to which you refer in your text on page 10. If the figure is no longer to be included as part of the submission please remove all reference to it within the text.

9. Please include captions for your Supporting Information files at the end of your manuscript, and update any in-text citations to match accordingly. Please see our Supporting Information guidelines for more information: http://journals.plos.org/plosone/s/supporting-information.

10. We notice that your supplementary tables are included in the manuscript file. Please remove them and upload them with the file type 'Supporting Information'. Please ensure that each Supporting Information file has a legend listed in the manuscript after the references list.

11. If the reviewer comments include a recommendation to cite specific previously published works, please review and evaluate these publications to determine whether they are relevant and should be cited. There is no requirement to cite these works unless the editor has indicated otherwise.

[Note: HTML markup is below. Please do not edit.]

Reviewers' comments:

Reviewer's Responses to Questions

**Comments to the Author**

1. If the authors have adequately addressed your comments raised in a previous round of review and you feel that this manuscript is now acceptable for publication, you may indicate that here to bypass the “Comments to the Author” section, enter your conflict of interest statement in the “Confidential to Editor” section, and submit your "Accept" recommendation.

Reviewer #1: All comments have been addressed

Reviewer #6: (No Response)

**********

2. Is the manuscript technically sound, and do the data support the conclusions?

The manuscript must describe a technically sound piece of scientific research with data that supports the conclusions. Experiments must have been conducted rigorously, with appropriate controls, replication, and sample sizes. The conclusions must be drawn appropriately based on the data presented.

Reviewer #1: Yes

Reviewer #6: No

**********

3. Has the statistical analysis been performed appropriately and rigorously?

Reviewer #1: N/A

Reviewer #6: N/A

**********

4. Have the authors made all data underlying the findings in their manuscript fully available?

The PLOS Data policy requires authors to make all data underlying the findings described in their manuscript fully available without restriction, with rare exception (please refer to the Data Availability Statement in the manuscript PDF file). The data should be provided as part of the manuscript or its supporting information, or deposited to a public repository. For example, in addition to summary statistics, the data points behind means, medians and variance measures should be available. If there are restrictions on publicly sharing data—e.g. participant privacy or use of data from a third party—those must be specified.

Reviewer #1: No

Reviewer #6: Yes

**********

5. Is the manuscript presented in an intelligible fashion and written in standard English?

PLOS ONE does not copyedit accepted manuscripts, so the language in submitted articles must be clear, correct, and unambiguous. Any typographical or grammatical errors should be corrected at revision, so please note any specific errors here.

Reviewer #1: Yes

Reviewer #6: Yes

**********

6. Review Comments to the Author

Please use the space provided to explain your answers to the questions above. You may also include additional comments for the author, including concerns about dual publication, research ethics, or publication ethics. (Please upload your review as an attachment if it exceeds 20,000 characters)

Reviewer #1: This manuscript presents a timely and relevant synthesis of the complex roles of steroid hormones and their receptors in cancer biology. The topic is important and aligns well with current directions in precision oncology, particularly in understanding hormone-driven malignancies. The effort to integrate molecular mechanisms with epidemiological and clinical insights is commendable. However, several key areas require further clarification and strengthening before the manuscript can be considered for publication.

Major Comments

• Clarity of Study Design (Narrative vs Systematic Review):

The manuscript alternates between describing itself as a “mini-review” and implying elements of a systematic review.

Please clearly define the review type (e.g., narrative review, scoping review, or systematic review).

If systematic elements are retained, include:

Search strategy (databases, keywords, time frame)

Inclusion/exclusion criteria

Study selection process (e.g., PRISMA flow diagram)

• Methodological Transparency:

o The literature search methodology lacks sufficient detail.

o A dedicated Methods section should be included even for a narrative review to enhance reproducibility and transparency.

• Critical Appraisal of Literature:

o The manuscript currently summarizes findings effectively but lacks critical evaluation.

o Strengthen the discussion by:

Highlighting conflicting findings across studies

Discussing study limitations (e.g., sample size, bias, population differences)

Identifying gaps in current knowledge

• Data Availability Statement:

o Although this is a review, PLOS ONE requires clarity regarding data sources.

o Include a statement specifying that all data are derived from published literature and provide supplementary material if applicable.

• Limitations Section:

o A dedicated section outlining the limitations of the review itself is needed.

o This should include:

Potential publication bias

Database selection limitations

Language restrictions (if any)

Minor Comments

• Consistency in Terminology:

o Ensure consistent use of terms such as “steroid hormones,” “sex hormones,” and “hormone-related receptors.”

• Language and Readability:

o Some sentences are overly complex and may reduce readability.

o Consider simplifying long sentences, especially in the Introduction and Discussion.

• Abbreviations:

o Ensure all abbreviations are defined at first mention (e.g., ER, PR, AR).

• Figures and Tables:

o Table 1 improvement is noted; however:

Ensure all entries are referenced

Consider adding a brief explanatory legend

o Figures illustrating hormone-receptor signaling pathways would enhance clarity.

Reviewer #6: The manuscript identifies several phytocompounds from S. barbata targeting breast cancer (BC) oncogenesis pathways using computational approaches. In particular, apigenin is highlighted as a potential candidate for modulating cancer signaling pathways. However, the conclusions are mainly based on in silico analyses, and further experimental validation is needed to confirm these findings.

The language in this manuscript is generally correct, but it is sometimes difficult to follow. The manuscript is also overly long and lacks a clear focus on the main points. In addition, some figures are of poor resolution (e.g., Fig. 1) and should be improved.

**********

7. PLOS authors have the option to publish the peer review history of their article (what does this mean?). If published, this will include your full peer review and any attached files.

If you choose “no”, your identity will remain anonymous but your review may still be made public.

**Do you want your identity to be public for this peer review?**  For information about this choice, including consent withdrawal, please see our Privacy Policy.

Reviewer #1: **Yes:**  Saheed Ayodeji Adekola

Reviewer #6: No

**********

[NOTE: If reviewer comments were submitted as an attachment file, they will be attached to this email and accessible via the submission site. Please log into your account, locate the manuscript record, and check for the action link "View Attachments". If this link does not appear, there are no attachment files.]

To ensure your figures meet our technical requirements, please review our figure guidelines: https://journals.plos.org/plosone/s/figures

You may also use PLOS’s free figure tool, NAAS, to help you prepare publication quality figures: https://journals.plos.org/plosone/s/figures#loc-tools-for-figure-preparation.

NAAS will assess whether your figures meet our technical requirements by comparing each figure against our figure specifications.

# Author comment: Author response to Decision Letter 4

**DOI:** 10.1371/journal.pone.0338874.r008

9 May 2026

Dear Editor,

Thank you for the editor's decision letter dated on April 09, 2026. Enclosed within this submission is our point-by-point responses to the comments raised by both reviewers/editors. We would like to take this opportunity to express our sincere thanks to the expert reviewers/editors who identified several areas in our manuscript that were needed corrections as well as modifications. We also would like to cordially thank you for allowing us the change to resubmit a revised version of the manuscript.

We have revised and updated the manuscript based on suggestions from the reviewers, incorporating several modifications. Please find all changes highlighted in RED color fonts in the revised manuscript. We also have provided a clean manuscript for your kind perusal.

Attachment Submitted filename: Response to Reviewers.pdf

# Peer review: Decision Letter 4

**DOI:** 10.1371/journal.pone.0338874.r009

**Authors:** Zhiling Yu, Zhiling Yu, Zhiling Yu, Zhiling Yu, Zhiling Yu

5 Jun 2026

In Silico Screening and Molecular Analyses Identify Apigenin from Scutellaria barbata as a Potent AKT1 Inhibitor in Breast Cancer

PONE-D-25-63895R4

Dear Author,

We’re pleased to inform you that your manuscript has been judged scientifically suitable for publication and will be formally accepted for publication once it meets all outstanding technical requirements.

Within one week, you’ll receive an e-mail detailing the required amendments. When these have been addressed, you’ll receive a formal acceptance letter and your manuscript will be scheduled for publication.

An invoice will be generated when your article is formally accepted. Please note, if your institution has a publishing partnership with PLOS and your article meets the relevant criteria, all or part of your publication costs will be covered. Please make sure your user information is up-to-date by logging into Editorial Manager at Editorial Manager® and clicking the ‘Update My Information' link at the top of the page. For questions related to billing, please contact billing support.

If your institution or institutions have a press office, please notify them about your upcoming paper to help maximize its impact. If they’ll be preparing press materials, please inform our press team as soon as possible -- no later than 48 hours after receiving the formal acceptance. Your manuscript will remain under strict press embargo until 2 pm Eastern Time on the date of publication. For more information, please contact onepress@plos.org.

Kind regards,

Editor

PLOS One

Additional Editor Comments (optional):

The authors have comprehensively addressed all previous concerns from the reviewers. The manuscript now satisfies PLOS ONE’s publication criteria and is deemed acceptable for publication.

Reviewers' comments:

Reviewer's Responses to Questions

**Comments to the Author**

1. If the authors have adequately addressed your comments raised in a previous round of review and you feel that this manuscript is now acceptable for publication, you may indicate that here to bypass the “Comments to the Author” section, enter your conflict of interest statement in the “Confidential to Editor” section, and submit your "Accept" recommendation.

Reviewer #7: All comments have been addressed

**********

2. Is the manuscript technically sound, and do the data support the conclusions?

The manuscript must describe a technically sound piece of scientific research with data that supports the conclusions. Experiments must have been conducted rigorously, with appropriate controls, replication, and sample sizes. The conclusions must be drawn appropriately based on the data presented.

Reviewer #7: Yes

**********

3. Has the statistical analysis been performed appropriately and rigorously?

Reviewer #7: N/A

**********

4. Have the authors made all data underlying the findings in their manuscript fully available?

The PLOS Data policy requires authors to make all data underlying the findings described in their manuscript fully available without restriction, with rare exception (please refer to the Data Availability Statement in the manuscript PDF file). The data should be provided as part of the manuscript or its supporting information, or deposited to a public repository. For example, in addition to summary statistics, the data points behind means, medians and variance measures should be available. If there are restrictions on publicly sharing data—e.g. participant privacy or use of data from a third party—those must be specified.

Reviewer #7: Yes

**********

5. Is the manuscript presented in an intelligible fashion and written in standard English?

PLOS ONE does not copyedit accepted manuscripts, so the language in submitted articles must be clear, correct, and unambiguous. Any typographical or grammatical errors should be corrected at revision, so please note any specific errors here.

Reviewer #7: Yes

**********

6. Review Comments to the Author

Please use the space provided to explain your answers to the questions above. You may also include additional comments for the author, including concerns about dual publication, research ethics, or publication ethics. (Please upload your review as an attachment if it exceeds 20,000 characters)

Reviewer #7: (No Response)

**********

7. PLOS authors have the option to publish the peer review history of their article (what does this mean?). If published, this will include your full peer review and any attached files.

If you choose “no”, your identity will remain anonymous but your review may still be made public.

**Do you want your identity to be public for this peer review?**  For information about this choice, including consent withdrawal, please see our Privacy Policy.

Reviewer #7: No

**********

# Editor report: Acceptance letter

**DOI:** 10.1371/journal.pone.0338874.r010

**Authors:** Zhiling Yu, Zhiling Yu, Zhiling Yu, Zhiling Yu, Zhiling Yu

PONE-D-25-63895R4

PLOS One

Dear Dr. Hoque,

I'm pleased to inform you that your manuscript has been deemed suitable for publication in PLOS One. Congratulations! Your manuscript is now being handed over to our production team.

At this stage, our production department will prepare your paper for publication. This includes ensuring the following:

* All references, tables, and figures are properly cited

* All relevant supporting information is included in the manuscript submission,

* There are no issues that prevent the paper from being properly typeset

You will receive further instructions from the production team, including instructions on how to review your proof when it is ready. Please keep in mind that we are working through a large volume of accepted articles, so please give us a few days to review your paper and let you know the next and final steps.

Lastly, if your institution or institutions have a press office, please let them know about your upcoming paper now to help maximize its impact. If they'll be preparing press materials, please inform our press team within the next 48 hours. Your manuscript will remain under strict press embargo until 2 pm Eastern Time on the date of publication. For more information, please contact onepress@plos.org.

You will receive an invoice from PLOS for your publication fee after your manuscript has reached the completed accept phase. If you receive an email requesting payment before acceptance or for any other service, this may be a phishing scheme. Learn how to identify phishing emails and protect your accounts at https://explore.plos.org/phishing.

If we can help with anything else, please email us at customercare@plos.org.

Thank you for submitting your work to PLOS ONE and supporting open access.

Kind regards,

PLOS ONE Editorial Office Staff

on behalf of

Dr. Zhiling Yu

Academic Editor

PLOS One
